# Hypoxia-induced Downregulation of SRC-3 Suppresses Trophoblastic Invasion and Migration Through Inhibition of the AKT/mTOR Pathway: Implications for the Pathogenesis of Preeclampsia

**DOI:** 10.1038/s41598-019-46699-3

**Published:** 2019-07-17

**Authors:** Chengjin He, Nan Shan, Ping Xu, Huisheng Ge, Yu Yuan, Yangming Liu, Pu Zhang, Li Wen, Fumei Zhang, Liling Xiong, Chuan Peng, Hongbo Qi, Chao Tong, Philip N. Baker

**Affiliations:** 1 Department of Obstetrics, The First Affiliated Hospital of Chongqing Medical University, Chongqing, 400016 China; 2 International Collaborative Joint Laboratory of Reproduction and Development, Ministry of Education of China, Chongqing Medical University, Chongqing, 400016 China; 3 State Key Laboratory of Maternal and Fetal Medicine of Chongqing Municipality, The First Affiliated Hospital of Chongqing Medical University, Chongqing, 400016 China; 4 College of Pharmacy, Chongqing Medical University, Chongqing, 400016 China; 5 Liggins Institute, University of Auckland, Auckland, 1142 New Zealand; 6 College of Life Sciences, University of Leicester, Leicester, LE1 7RH UK

**Keywords:** Non-coding RNAs, Reproductive disorders

## Abstract

Preeclampsia (PE) is characterized by poor placentation, consequent on aberrant extravillous trophoblast (EVT) cell function during placental development. The SRC family of proteins is important during pregnancy, especially SRC-3, which regulates placental morphogenesis and embryo survival. Although SRC-3 expression in mouse trophoblast giant cells has been documented, its role in the functional regulation of extravillous trophoblasts and the development of PE remains unknown. This study found that SRC-3 expression was significantly lower in placentas from PE pregnancies as compared to uncomplicated pregnancies. Additionally, both CoCl_2_-mimicked hypoxia and suppression of endogenous SRC-3 expression by lentivirus short hairpin RNA attenuated the migration and invasion abilities of HTR-8/SVneo cells. Moreover, we demonstrated that SRC-3 physically interacts with AKT to regulate the migration and invasion of HTR-8 cells, via the AKT/mTOR pathway. We also found that the inhibition of HTR-8 cell migration and invasion by CoCl_2_-mimicked hypoxia was through the SRC-3/AKT/mTOR axis. Our findings indicate that, in early gestation, accumulation of HIF-1α inhibits the expression of SRC-3, which impairs extravillous trophoblastic invasion and migration by directly interacting with AKT. This potentially leads to insufficient uterine spiral artery remodeling and placental hypoperfusion, and thus the development of PE.

## Introduction

Preeclampsia (PE) is a serious pregnancy-specific complication that affects 3–8% of all pregnancies and is the leading cause of maternal and fetal morbidity/mortality^1,2^. It has long been believed that the development of PE stems from defective placentation, however, the precise etiology of PE remains poorly understood^3,4^. Trophoblast cells are at the interface between the embryonic and maternal vascular systems. The migration and invasion capacity of these cells play a crucial role in placentation, embryo implantation, and other important functions. Abnormalities in trophoblast cell function during placental development result in poor placentation and fetal growth restriction and are associated with PE^5,6^. In addition, accumulating evidence suggests that hypoxia is a critical event during the development of PE, one that is associated with abnormal differentiation of trophoblasts and increases in proinflammatory cytokine expression levels and oxidative stress^7–9^. Previous studies have shown that hypoxia leads to defective trophoblast invasion; this has been attributed to several factors, however, the underlying mechanism has yet to be fully elucidated^10–13^.

The p160 steroid receptor co-activator (SRC) family member SRC-3 (also known as NCOA3, AIB1, ACTR, pCIP, RAC3, and TRAM-1) is an oncogene that has been reported to be amplified and/or overexpressed in a variety of tumors, including ovarian cancer, esophageal cancer, colorectal cancer, and breast cancer^14–16^. Recently, studies have reported that SRC-3 participates in tumorigenesis by regulating the proliferation and invasion of cancer cells^15,17^. Animal studies have revealed that overexpression of SRC-3 in transgenic mice promotes the development of breast cancer^18^. Trophoblasts share a number of similarities with cancer cells and trophoblast tissue has been defined as a ‘pseudo-malignant’ or ‘physiological metastasis’; trophoblasts may thus be associated with similar expression patterns of SRC-3^19^. Additionally, the SRC family (including SRC-1, SRC-2, and SRC-3) is expressed in the human placenta; as its expression initially increases following conception and continually increases during gestation, it is considered critical for maintaining pregnancy^20^. A previous study of SRC-3 knockout mice indicated that the loss of SRC-3 in mice placenta led to reduced fetal capillaries and maternal blood sinusoids in the labyrinth area of these mice as compared to wild-type mice^21^. Our previous work suggested that SRC-3 influences the migration and tube formation of endothelial cells, which is related to vascular endothelial dysfunction and recognized features of PE^22^. SRC-3 was also detected in trophoblast giant cells^21^, which are widely accepted as mediating the invasion of the endometrium during mice placentation^23,24^. However, the role of SRC-3 in the regulation of trophoblastic invasion and migration remains unknown.

In the present study, SRC-3-deficient trophoblast cells and a CoCl_2_-mimicked hypoxia model were used to investigate whether SRC-3 impacts the proliferation, migration, and invasion of trophoblast cells, as well as to determine the relevance of SRC-3 to the subsequent development of PE.

## Results

### Clinical characteristics

The clinical characteristics of the study subjects are shown in Table 1. The age and parity were similar between the PE and the uncomplicated pregnancy groups. Women suffering from PE had significantly higher antenatal body mass index (BMI), systolic blood pressure, diastolic blood pressure, and proteinuria, but mean gestational age at birth, neonatal birth weight, and placental weight were lower, as compared to uncomplicated pregnant women.

**Table 1:** Clinical characteristics of the human subjects.

Category	Control (n = 25)	Preeclampsia (n = 25)
Age (years)	29.1 ± 2.83	29.4 ± 2.59
Gestational age at birth (weeks)	40.07 ± 0.44	36.86 ± 1.60^***^
Body mass index (BMI; kg/m^2^)	27.98 ± 1.42	30.33 ± 2.31^***^
Gravidity	1.90 ± 0.68	1.95 ± 0.58
Proteinuria (g/24 h)	0.05 ± 0.01	2.69 ± 0.07^***^
Systolic blood pressure (mmHg)	110.8 ± 6.65	158.5 ± 8.67^***^
Diastolic blood pressure (mmHg)	73.7 ± 7.25	106.4 ± 8.24^***^
Neonatal birth weight (g)	3402 ± 313.53	2640 ± 121.52^***^
Neonatal birth length (cm)	50.01 ± 0.99	47.24 ± 0.92^***^
Placental weight (g)	555.5 ± 28.37	473.4 ± 25.44^***^

Body mass index (BMI) formula: weight (kg)/height^2^ (m^2^).

*p < 0.05, **p < 0.01, ***p < 0.001.

### SRC-3/p-AKT/p-mTOR expression is downregulated in PE human placentas

**Figure 1:**
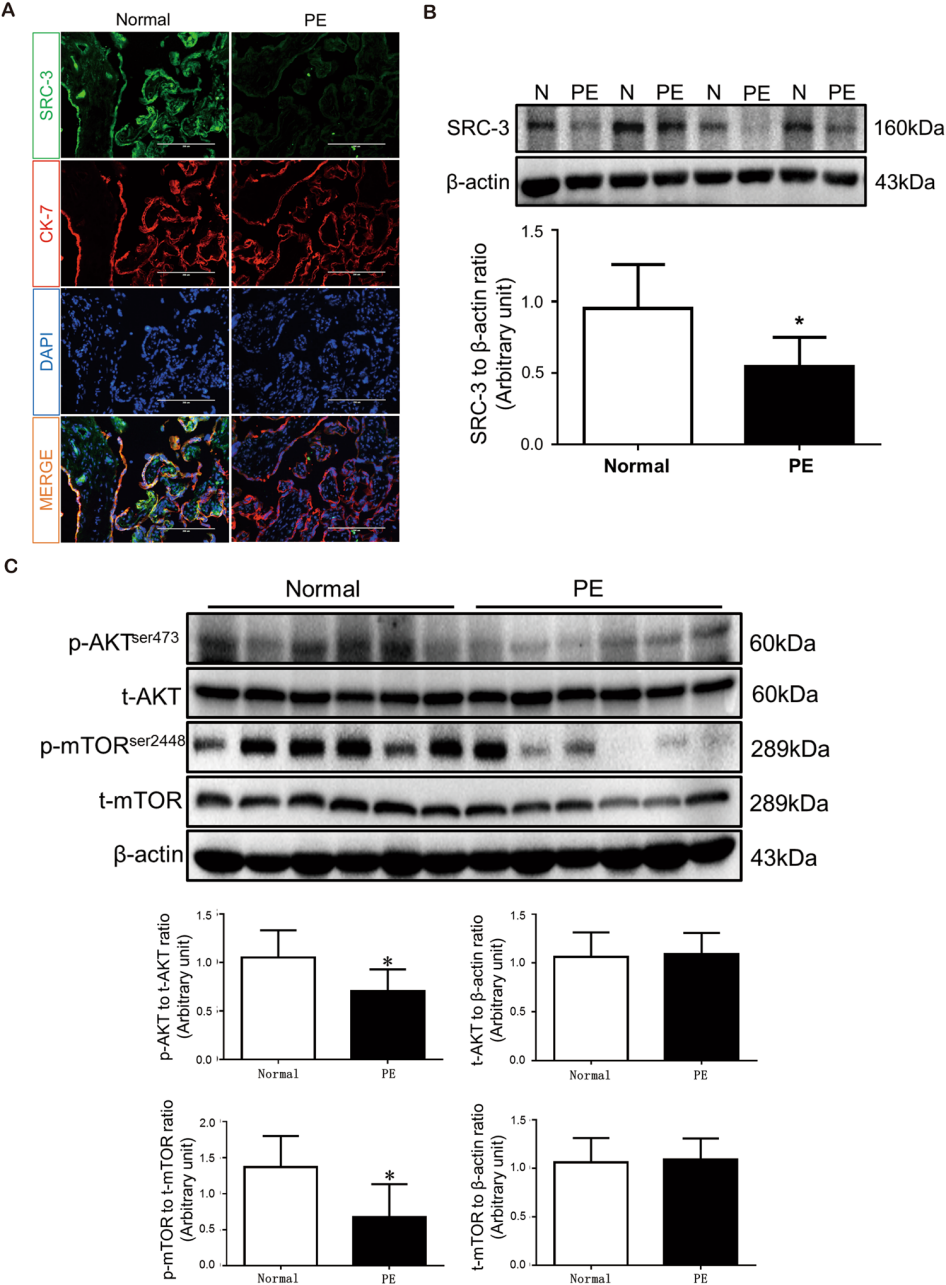
SRC-3 expression pattern and AKT/mTOR signaling pathway component expression in normal and PE human placentas. (**A**) IF staining of SRC-3 (green) and CK7 (red) in frozen sections of human term-placentas; nuclei were counterstained by DAPI (blue). Scale bars: 200 μm. (**B**) Western blots of SRC-3 in human term-placentas, n = 5, *p < 0.05. (**C**) Western blots of AKT, p-AKT, mTOR, and p-mTOR protein expression in human term-placentas. β-actin served as a loading control, n = 6, *p < 0.05. All experiments were repeated at least three times.

To determine the involvement of SRC-3 in PE development, we first determined the expression pattern of SRC-3 in human placentas by immunofluorescence (IF) staining. As shown in Fig. 1A, SRC-3 was ubiquitously expressed in placental tissue and was primarily found in trophoblasts. Intriguingly, the expression level of SRC-3 was significantly lower in PE placentas than in placentas from uncomplicated pregnancies. Western blotting demonstrated that SRC-3 protein levels were reduced by 43% in PE placentas (Fig. 1B), while the phosphorylation levels of AKT and mTOR were also significantly reduced in PE human placentas as compared to placentas from uncomplicated pregnancies (Fig. 1C).

### Inhibition of SRC-3 expression does not alter trophoblast viability

**Figure 2:**
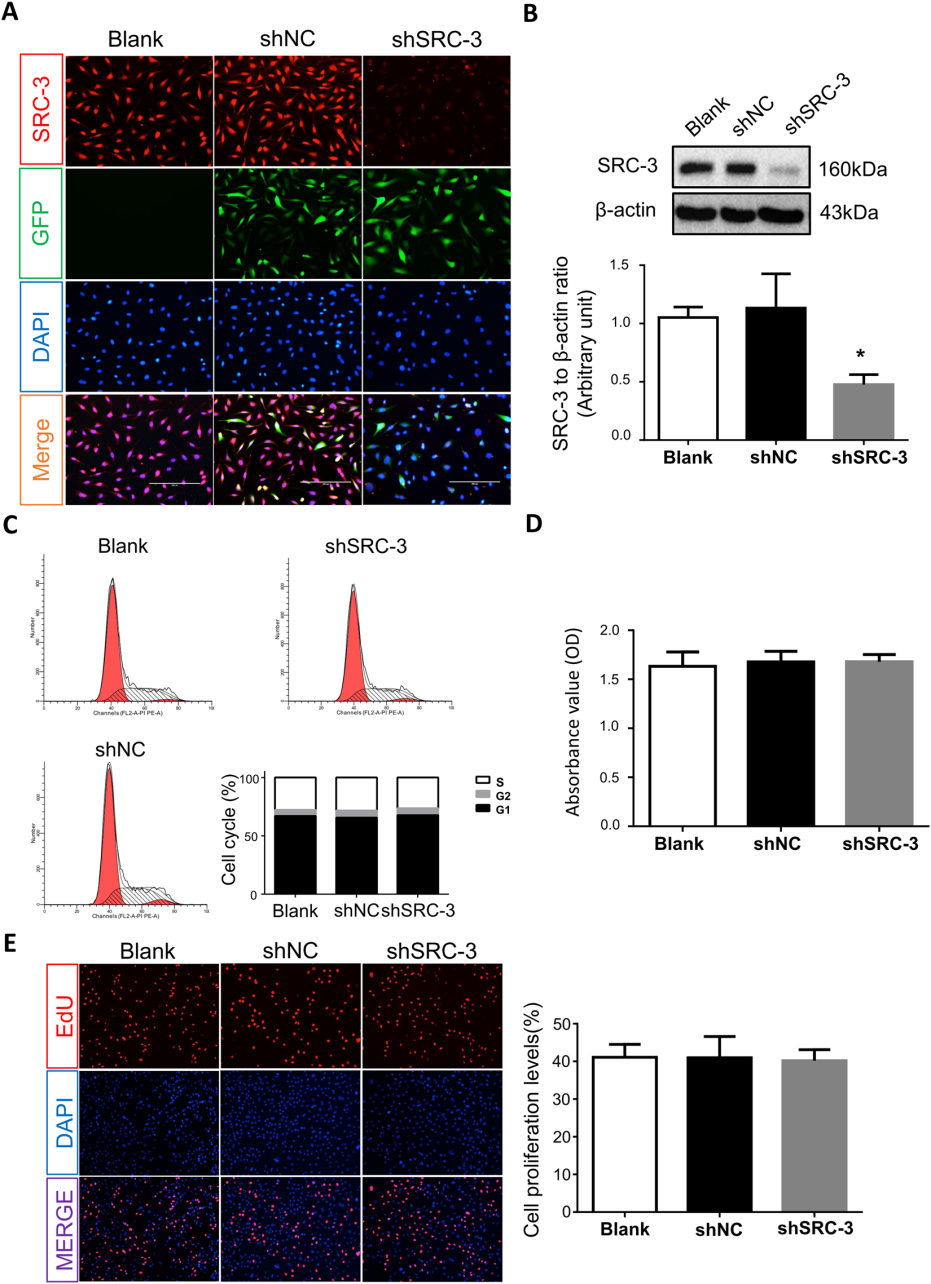
SRC-3 knockdown in HTR-8/SVneo cells. Wild-type, GFP-labeled shSRC-3, or shNC-transfected HTR-8/SVneo cells were subjected to: (**A**) IF staining for SRC-3 (red); transfected cells were recognized by GFP staining (green), while nuclei were counterstained with DAPI (blue). (**B**) SRC-3 protein expression levels were confirmed by western blot analysis; β-actin served as a loading control, n = 3, *p < 0.05; Cell proliferation was assessed by (**C**) flow cytometry, (**D**) CCK-8 staining, and (**E**) EdU staining. All experiments were repeated three times.

To further investigate the role of SRC-3 in trophoblast cells, SRC-3 expression levels in human HTR-8/SVneo trophoblast cells were reduced by short hairpin RNA (shRNA) transfection. IF staining demonstrated that SRC-3 levels in HTR-8/SVneo cells were markedly reduced by shSRC-3 transfection (Fig. 2A), and Western blotting confirmed that SRC-3 expression was reduced by nearly 50% (Fig. 2B).

Since SRC-3 has been reported to be involved in the regulation of cell growth and proliferation in various cancer cells^14,15,25^, we next assessed the effects of SRC-3 on trophoblast proliferation. Flow cytometry analysis revealed that downregulation of SRC-3 by shRNA did not result in cell-cycle arrest in HTR8/SVneo cells (Fig. 2C). Consistent with this finding, neither CCK-8 nor EdU staining assays demonstrated that the proliferation of trophoblasts was influenced by SRC-3 inhibition (Fig. 2D,E), which implies that SRC-3 may not be critical for DNA replication in HTR8/SVneo cells. SRC-3-knockdown (KD) cells demonstrated comparable proliferation rates to blank control and scramble shRNA (shNC) transfected cells (Fig. 2C), further indicating that SRC-3 is not a determinant of proliferation in trophoblasts.

### SRC-3 regulates migration and invasion of HTR8/SVneo cells through the AKT/mTOR signaling pathway

**Figure 3:**
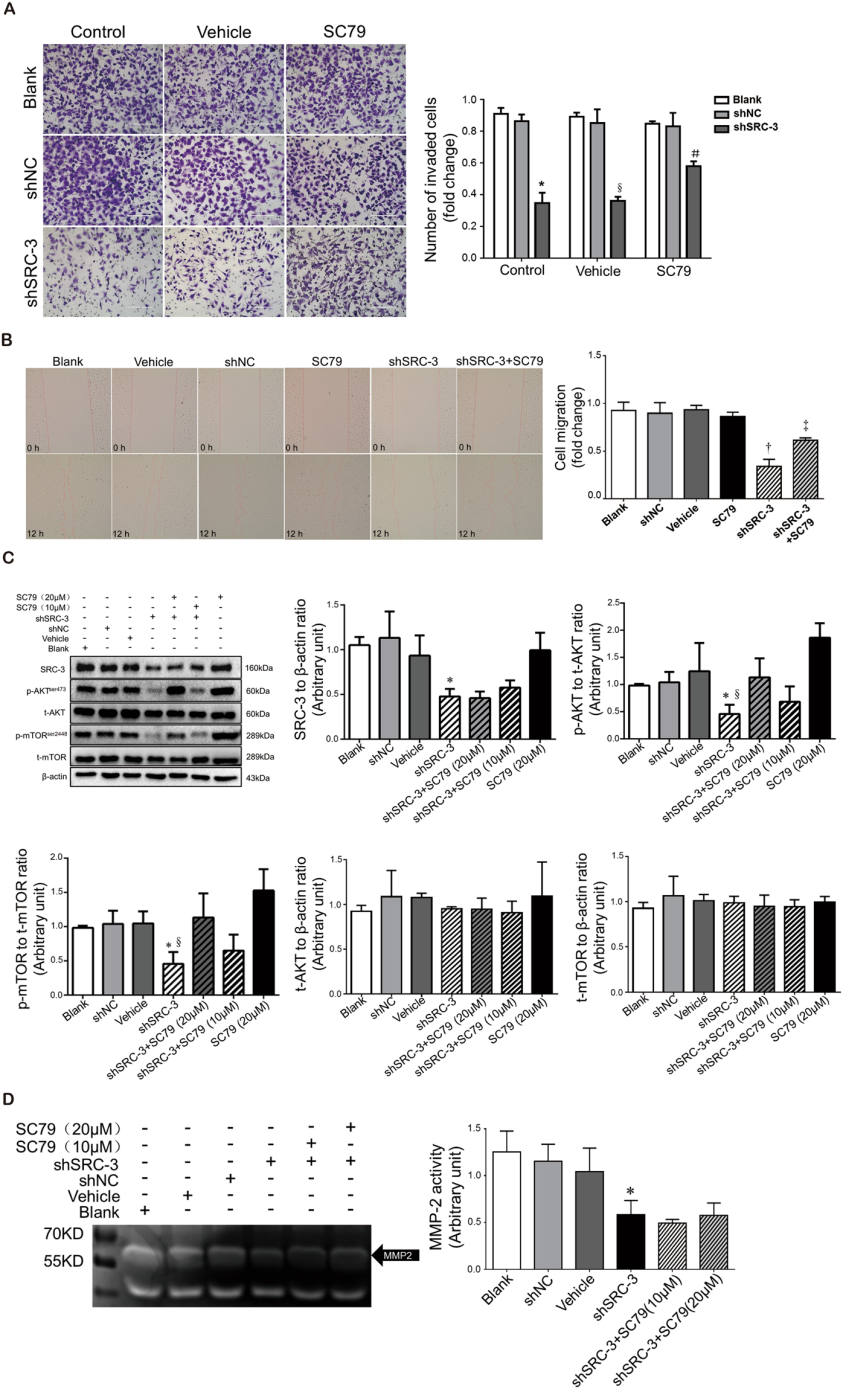
Downregulation of SRC-3 expression suppresses the migration and invasion of HTR8/SVneo cells through the AKT/mTOR signaling pathway. Non-transfected and shNC- or shSRC-3-transfected HTR8/SVneo cells were treated with 20 μM SC79 or 0.1% DMSO (vehicle control) and subjected to: (**A**) Matrigel transwell assays. Invaded cells were stained and counted after 24 h. n = 3, *p < 0.001 vs. Blank control and shNC control, §p < 0.001 vs. Blank vehicle & shNC vehicle, #p < 0.01 vs. shSRC-3 control & shSRC-3 vehicle. Scale bars: 200 μm. (**B**) Wound-healing assays. Images were taken at 0 h and 12 h of treatment. Quantification of the areas of migration is shown in the bar graph. n = 3, †p < 0.001 vs. Blank & shNC, ‡p < 0.001 vs. shSRC-3. (**C**) Western blotting of SRC-3, AKT, p-AKT, mTOR, and p-mTOR in the aforementioned groups of cells, and a lower dose of SC79 (10 μM) treatment group was added, n = 3, *p < 0.01 vs. shNC, §p < 0.01 vs. shSRC-3 + SC79 (20 μM). (**D**) Gelatin zymography of MMP-2 activity in the culture medium of cells in the presence of vehicle (0.1% DMSO), 10 μM SC79, or 20 μM SC79 over 24 h, n = 3, *p < 0.05 vs. shSRC-3. Scale bars: 400 μm (**D**). All experiments were repeated three times.

Migration and invasion of trophoblast cells play a crucial role in placentation, and downregulation of AKT/mTOR signaling has been correlated with cancer cell migration and invasion^26–28^. We thus evaluated the role of SRC-3 in modulating the migratory and invasive capabilities of trophoblast cells. Matrigel-based invasion assays demonstrated that inhibition of SRC-3 expression significantly diminished the invasiveness of HTR8/SVneo cells (Fig. 3A); the loss of invasiveness was largely rescued following treatment with SC79, an AKT activator. Similarly, wound-healing experiments determined that the migratory capability of trophoblast cells was suppressed in the presence of shSRC-3 (Fig. 3B) but could be enhanced by SC79 treatment. Western blotting results showed that the knockdown of SRC-3 significantly reduced p-AKT and p-mTOR levels, while the activation of AKT and mTOR was restored by SC79 treatment in a dose-dependent manner (Fig. 3C). Furthermore, gelatin zymography illustrated that SRC-3 interference significantly decreased MMP-2 activity in HTR8/SVneo cells; the loss in MMP-2 activity could not be restored by either low- or high-dose SC79 treatment (Fig. 3D). Taken together, these results indicate that SRC-3 regulates the migration and invasion of HTR8/SVneo cells via the AKT/mTOR pathway, independent of MMP-2.

### SRC-3 physically interacts with AKT in HTR-8/SVneo

**Figure 4:**
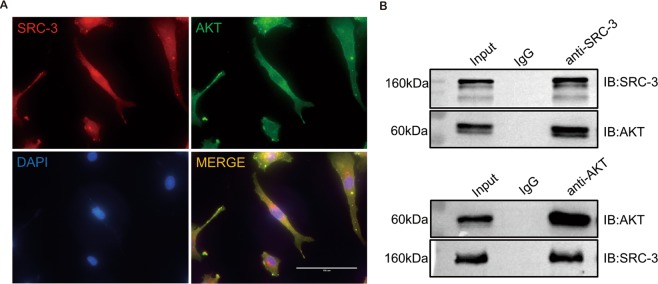
SRC-3 directly interacts with AKT in HTR-8/SVneo cells. (**A**) Representative images of IF staining of SRC-3 (red) and AKT (green) in HTR-8/SVneo cells. Nuclei were counterstained with DAPI (blue). Scale bars: 100 μm. (**B**) Co-IP of AKT with SRC-3 in HTR8/SVneo cells. All experiments were repeated three times.

The family of SRC proteins contain a bHLH/PAS domain, which is normally involved in protein–protein interactions, indicating that SRC proteins may regulate downstream effectors through physical interactions. Indeed, it has been reported that SRC-3 binds the C-terminal activation domain of myocardin and enhances myocardin-mediated transcriptional activation of its downstream factors^29,30^. Therefore, we next investigated whether the regulatory effects of SRC-3 on trophoblast cell invasion are mediated by direct interactions between SRC-3 and AKT. First, IF staining showed that AKT was ubiquitously expressed in HTR8/SVneo cells, whereas SRC-3 was predominantly localized in the nucleus (Fig. 4A). The co-localization of SRC-3 and AKT in the nucleus suggests that SRC-3 might bind to AKT and thus modulate the transcriptional activity of AKT in trophoblast cells. To further validate the putative physical interaction between SRC-3 and AKT, reciprocal co-immunoprecipitation (IP) experiments were performed; the results clearly show that SRC-3 binds to AKT in trophoblasts (Fig. 4B).

### Hypoxia suppresses the migration and invasion of HTR8/SVneo cells through inhibition of the SRC-3/AKT/mTOR signaling axis

**Figure 5:**
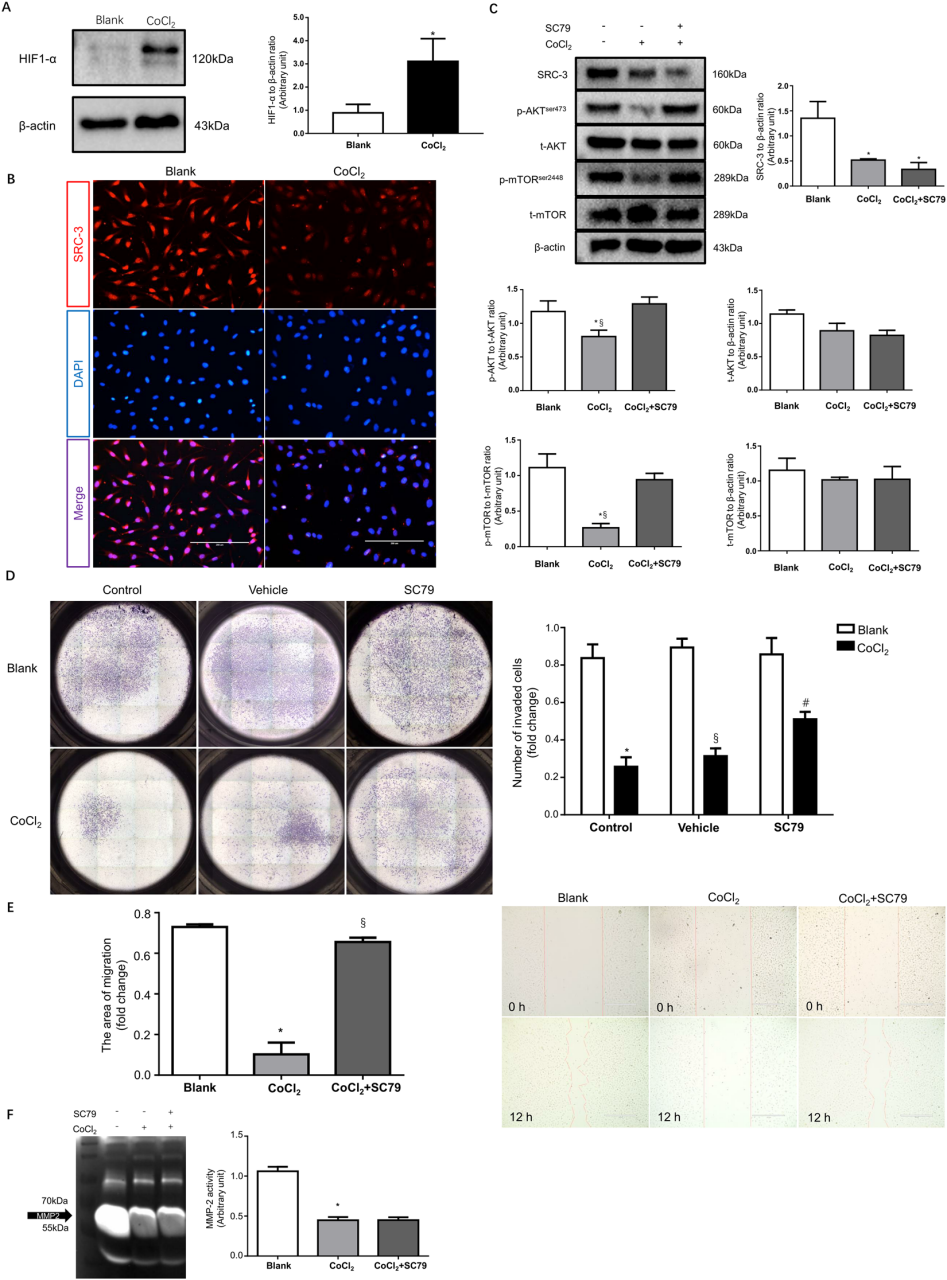
Hypoxia impairs the migration and invasion of HTR8/SVneo cells through inhibition of the SRC-3/AKT/mTOR signaling axis. After 48 h of 250 μM CoCl_2_ treatment, HTR8/SVneo cells were subjected to: (**A**) Western blot analysis of HIF1-α. (**B**) IF staining of SRC-3, scale bar: 200 μm. (**C**) Western blot analysis of SRC-3, AKT, p-AKT, mTOR, and p-mTOR levels in HTR8/SVneo cells in the presence of 250 μM CoCl_2_ and/or 20 μM SC79. The blank control was also included, n = 3, *p < 0.05 vs. Blank, §p < 0.05 vs. CoCl_2_ + SC79. (**D**) HTR8/SVneo cells subjected to Matrigel transwell assays in the presence of vehicle (0.1% DMSO), 250 μM CoCl_2_, and/or 20 μM SC79. Invaded cells were counted after 24 h. n = 3, *p < 0.001 vs. Blank control, §p < 0.001 vs. Blank vehicle, #p < 0.01 vs. CoCl_2_ control. (**E**) Wound-healing assays for HTR8/SVneo cells in the presence of 250 μM CoCl_2_ and/or 20 μM SC79. Images were taken at 0 h and after 12 h of treatment. The areas of migration were quantified in the bar graph. n = 3, *p < 0.001 vs. Blank, §p < 0.001 vs. CoCl_2_. (**F**) Gelatin zymography of MMP-2 activity in culture medium of HTR8/SVneo cells in the presence of 250 μM CoCl_2_ and/or 20 μM SC79, *p < 0.001 vs. Blank. All experiments were repeated three times.

To explore the mechanism underlying SRC-3 downregulation in PE placenta, HTR8/SVneo cells were subjected to cobalt chloride (CoCl_2_) treatment to establish an *in vitro* trophoblast hypoxia model. The expression of HIF-1 was significantly elevated by CoCl_2_, confirming that a hypoxia response had been induced in HTR8/SVneo cells (Fig. 5A). Subsequently, IF staining showed that CoCl_2_-mimicked hypoxia dramatically attenuated the expression of SRC-3 in HTR8/SVneo cells (Fig. 5B); this was further validated by Western blotting (Fig. 5C). In addition, CoCl_2_ treatment significantly decreased the phosphorylation of AKT and mTOR in trophoblasts, but it did not change the total protein expression levels of AKT and mTOR (Fig. 5C). Although SC79 treatment almost fully alleviated the dephosphorylation of AKT and mTOR induced by CoCl_2_, it failed to rescue SRC-3 expression in trophoblasts. These results indicate that hypoxia impairs SRC-3 expression in trophoblasts, which subsequently results in the inhibition of the AKT/mTOR pathway. Furthermore, CoCl_2_ treatment significantly suppressed the invasion of HTR8/SVneo cells; this suppression was blunted in the presence of SC79 (Fig. 5D). Similarly, trophoblasts treated with CoCl_2_ were associated with a reduction in migration, which could be blocked by SC79 treatment (Fig. 5E). However, SC79 treatment failed to rescue MMP-2 inhibition in HTR-8 cells resulting from CoCl_2_ treatment (Fig. 5F). These findings are consistent with our observations in PE placentas, and suggest that CoCl_2_-induced downregulation of SRC-3 compromises trophoblastic invasion and migration through the AKT/mTOR signaling pathway independent of MMP-2.

## Discussion

SRC-3 has been intensively studied in several malignant tumors and is thought to be an oncogene that promotes carcinogenesis^15,31^. Since first trimester trophoblasts show considerable similarities to malignant cells, they have been suggested to utilize similar mechanisms for the regulation of proliferation, migration, and invasion^32^. Accumulating evidence has suggested that SRC-3 might play a pivotal role in maintaining pregnancy^20,21^. In this study, we demonstrated that PE placentas express lower levels of SRC-3 than placentas from uncomplicated pregnancies, which is consistent with a prior report^22^ and potentially implicates a loss of placental SRC-3 expression in the development of PE. It has been shown that SRC-3-knockout mice have smaller sinusoids in the labyrinth area^21^. In contrast, upregulation of SRC-3 promotes the proliferation and growth of tumor tissues in a broad spectrum of cancers^15,29^.

We utilized multiple approaches to evaluate the effects of SRC-3 on trophoblast proliferation. No marked differences were found between SRC-3-knockdown and wild-type HTR8/SVneo cells, despite AKT/mTOR being downregulated by knockdown of SRC-3. There are extensive cross-talk pathways between the PI3K/AKT/mTOR and the ERK-MAPK signaling pathways^33,34^. When any of these pathways are blocked, cross-inhibition and activation of compensatory signaling often occurs^35–37^. For example, it has been shown that blocking each pathway alone is less efficient in suppressing cancer cell proliferation than the combined inhibition of PI3K and MAPK^33,35,38^. Further investigation into the effects of SRC-3 on ERK signaling will be helpful to fully elucidate the role of SRC-3 in modulating the proliferation and viability of trophoblast cells.

SRC-3 has been reported to regulate cell invasion^15,16^, and as expected, repression of SRC-3 expression coincided with reduced invasion and migration of trophoblast cells. Migration- and invasion-related extracellular matrix (ECM) degradation requires the action of MMP-2^39,40^, and MMP-2 is regulated by PI3K/AKT signaling^26,27^. Therefore, we further studied whether the SRC-3-AKT signaling axis regulates trophoblast migration by modulating MMP-2 activity. MMP-2 activity was reduced in SRC-3-knockdown cells. Previous studies have further suggested that SRC-3 could be an upstream regulator of the PI3K signaling pathway^18,41,42^. Our data confirmed that SRC-3 expression is positively correlated with AKT and mTOR phosphorylation. Intriguingly, although SC79 treatment restored the p-mTOR levels, as well as the invasion and migration capabilities of SRC-3-KD trophoblast cells, it did not rescue MMP-2 activity, suggesting that the regulation of trophoblastic invasion and migration by the SRC-3-AKT-mTOR signaling axis is not mediated by MMP-2. However, SRC-3 is a polyomavirus enhancer activator 3 (PEA3) coactivator and in breast cancer cells SRC-3 forms complexes with PEA3 on MMP-2 promoters to enhance its expression^43^. Further studies in trophoblasts are required.

Since the SRC family contains a bHLH/PAS domain that mediates protein–protein interactions^29,30^, we speculated that SRC-3 might directly interact with AKT and regulate its activation in trophoblast cells. Indeed, our results are the first to confirm that SRC-3 binds to AKT; this may be critical for SRC-3/AKT signal transduction and the subsequent regulation of trophoblastic function.

In early gestation, trophoblast cells have to be resistant to a low oxygen intrauterine environment^44–46^. However, in placentas of pregnancies complicated by preeclampsia, low oxygen triggers a hypoxic response, which leads to an elevation of HIF-1α, a consequent reduction in trophoblast invasion, and placental hypoperfusion. The accumulation of HIF-1α in PE trophoblasts may be caused by various possible mechanisms, including an insufficient increase in placental oxygen tension after the 12^th^ week of gestation, catechol-O-methyl transferase (COMT) deficiency^47,48^, decreased expression of prolyl hydroxylase 2 (PHD-2), and/or other inhibitory regulators of HIF-1^49–51^.

We established a CoCl_2_-mimicked hypoxic trophoblast model and found that hypoxia reduced SRC-3 expression. Interestingly, although both hypoxia and SRC-3 knockdown lead to MMP-2 inhibition, the impaired trophoblastic invasion and migration due to hypoxia-induced SRC-3 downregulation is mediated by the AKT-mTOR pathway independent of MMP-2 activity. SRC-3 was initially discovered as a steroid receptor co-activator that could mediate chromatin remodeling and enhance receptor-dependent transcription by recruiting additional factors, such as acetyltransferases (e.g., CBP and p300) and methyltransferases (e.g., CARM1 and PRMT1)^52^. Thus, the involvement of these molecules in SRC-3-regulated trophoblastic function is worthy of further investigation.

Chen *et al*. demonstrated that in addition to trophoblast giant cells, SRC-3 is also highly expressed in layer III syncytiotrophoblasts, which are responsible for fetal-maternal nutrient exchange in the labyrinth^21^. As a result, SRC-3-KO mice are associated with a small labyrinth, reduced fetal vascular density, and dilated maternal blood sinuses^21^. In human placentas, villous trophoblast malfunction and deficient fetoplacental angiogenesis are associated with PE^53–55^. In addition to regulating extravillous trophoblast (EVT) invasion, SRC-3 may contribute to the development of PE by interfering with villi branching. Further investigation into both mechanisms could aid understanding of the role of SRC-3 in PE.

**Figure 6:**
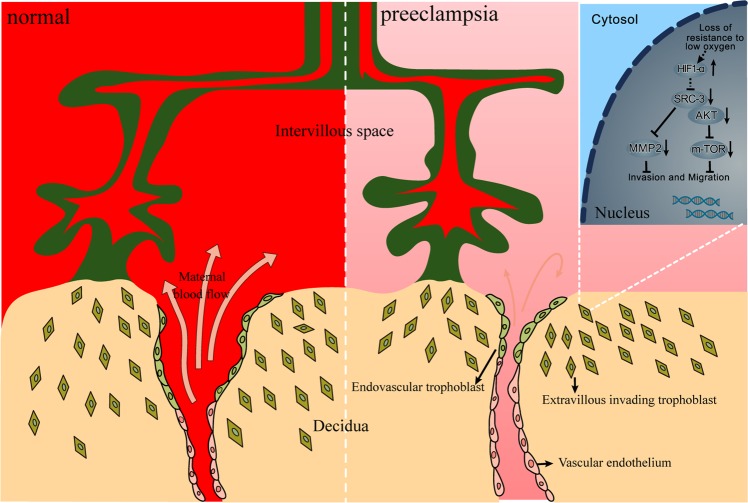
A working model of SRC-3/AKT/mTOR signaling in the regulation of trophoblastic invasion and PE development.

In conclusion, SRC-3 binds to AKT to activate the AKT/mTOR signaling pathway in EVT, which regulates cell migration and invasion independent of MMP-2 activity. Hypoxic response-induced SRC-3 downregulation may be an important pathologic mechanism underlying PE (Fig. 6). Our findings provide new insights into the etiology of PE and suggest potential targets for therapeutic intervention.

## Materials and Methods

### Patients and tissue sample collection

25 women whose pregnancies were complicated by PE and 25 normal pregnant women admitted to the Department of Obstetrics at The First Affiliated Hospital of Chongqing Medical University were recruited. PE was diagnosed according to the criteria of the American College of Obstetrics and Gynecology. Exclusion criteria included the presence of chronic hypertension, diabetes mellitus, renal diseases, or other metabolic diseases. This study was in accordance with the principles set out in the Declaration of Helsinki and was approved by the Ethics Committee of the First Affiliated Hospital of Chongqing Medical University. All participants provided informed consent. Placental tissues were collected immediately after cesarean section, washed in ice-cold 0.9% saline, and stored at −80 °C until further processing. Tissue collection processes and preparation for immunofluorescence were performed as described previously^22^.

### Cell culture and hypoxia treatment

HTR8/SVneo cells were kindly provided by Dr. Charles H. Graham (Kingston, ON, Canada). The HTR8/SVneo cells were maintained in RPMI-1640 (Gibco, Carlsbad, California, USA) supplemented with 10% fetal bovine serum (FBS; Gibco), at 37 °C in a humidified atmosphere with 5% CO_2_. To mimic hypoxia in trophoblasts, cells were treated with CoCl_2_ at a concentration of 250 µM for 48 h^56,57^. Cells were harvested using a commercial lysis buffer (Beyotime, Jiangsu, China), while culture medium was collected for zymography assays.

### SRC-3 knockdown by lentiviral shRNA

The target sequence of SRC-3 for constructing lentiviral shRNA was: shSRC-3: 5′-AGACTCCTTAGGACCGCTT-3′, shNC: 5′-TTCTCCGAACGTGTCACGT-3′. HTR8/SVneo cells were transfected with shRNA and shNC (GenePharma, Shanghai, China), according to the manufacturer’s protocol. At 24 h after transfection, 5 µg/ml puromycin was added for three days to screen SRC-3-KD cells.

### Immunofluorescence

Immunostaining was performed on frozen sections of human term placentas and HTR8/SVneo cells as described previously^22^. Briefly, the placental frozen sections and trophoblast cells were permeabilized with 0.2% Triton X-100 (Beyotime) and blocked with 1% bovine serum albumin (BSA) (Beyotime). The tissues and cells were then incubated with a primary antibody against SRC-3 (1:100, #5765), cytokeratin-7 (CK-7) (1:100, #4465) (Cell Signaling Technology, Danvers, MA, USA) at 4 °C overnight followed by incubation with the appropriate secondary antibody for 2 h (Santa Cruz Biotechnology, Dallas, TX, USA). The nuclei were counterstained by 4′, 6-diamidino-2-phenylindole (DAPI; Beyotime). Images were captured using a fluorescence microscope (Life Technologies, Waltham, MA, USA).

### Western blotting

Total protein in placental tissues or HTR8/SVneo cells were extracted with RIPA lysis buffer (Beyotime) and measured by bicinchoninic acid (BCA) protein assays (Beyotime). Twenty micrograms of total protein extracted from placental tissue or cell lysates were resolved by sodium dodecyl sulfate (SDS)-polyacrylamide gel electrophoresis followed by transfer to polyvinylidene difluoride (PVDF) membranes (Roche, Germany). These were further incubated with primary antibodies against SRC-3 (1:1000, #5765), p-AKT (Ser473) (1:1000, #9271), AKT (1:1000, #9272), mTOR (1:1000, #2983), or β-actin (1:1000, #3700), purchased from Cell Signaling Technology), or a primary antibody against p-mTOR (Ser2448) (1:1000; ab109268) purchased from Abcam (Cambridge, MA, USA). Subsequently, the membranes were incubated with a secondary antibody (1:5000) linked to horseradish peroxidase (Santa Cruz Biotechnology) and then enhanced chemiluminescence detection reagents (Millipore, Danvers, MA, USA) to detect the protein of interest. Identified bands were analyzed with a ChemiDoc image analyzer (Bio-Rad, Hercules, CA, USA).

### *In vitro* cell migration and invasion assays

Invasion assays were performed in 24-well plates with Matrigel (BD Biosciences, USA)-coated Trans well inserts (8.0 μm, Merck Millipore, Darmstadt, Germany) as described previously^58^, with the exception that 8 × 10^4^ cells were plated in the upper chamber of the membrane in these experiments.

The migratory ability of the HTR8/SVneo cells was determined by wound healing assay. In brief, cells were seeded in a 6-well plate and cultured to 80% confluence, and then a scratch on the cell monolayer was made using a pipette tip. The cells were then rinsed twice with fresh culture medium and allowed to stay in culture for another 24 h. Five fields were randomly selected and imaged at the beginning (0 h) and the end of the culture period (24 h). The number of invaded and migrated cells was counted under a microscope. All experiments were performed in triplicate.

### CCK-8 proliferation assay

Proliferation of HTR8/SVneo cells was measured with the use of a CCK-8 (Biotool, Houston, TX, USA) according to the manufacturer’s instructions. Briefly, 5 × 10^3^ cells were seeded in 96-well plates that contained RPMI-1640 and 10% FBS. Cell proliferation was examined 3 h after standard procedures. The absorbance at 450 nm was measured by a microplate reader (Thermo Fisher Scientific, Waltham, MA, USA). All experiments were repeated three times.

### Cell-cycle analysis

Several pretreated cells were seeded in a 6-well plate at a density of 8 × 10^5^ HTR8/SVneo cells per well. These cells were subsequently detached using trypsin and fixed with ice-cold 70% ethanol. The cells were then stained for cell-cycle analysis using a Coulter DNA-Prep Reagents Kit (Beckman Coulter, UK). Cellular DNA levels from each sample were determined with a FACScan flow cytometer (CytoFLEX, Beckman, China). All experiments were performed in triplicate.

### EdU staining

A total of 5 × 10^3^ HTR8/SVneo cells were seeded in 96-well plates and cultured to 80% confluence. Cells were treated with 25 µM of 5-ethynyl-2′-deoxyuridine (Cell-Light™ EdU Apollo®567 *In Vitro* Imaging Kit; Ribobio, Guangzhou, China) for 2 h at 37 °C, and then cells were fixed in 4% paraformaldehyde (PFA). Subsequently, the cells were permeabilized in 0.5% Triton-X for 10 min and exposed to 1X Apollo reaction cocktail (Cell-Light™ EdU Apollo®567 *In Vitro* Imaging Kit; Ribobio) for 30 min. Finally, cell nuclei were counterstained with Hoechst 33342 for 30 min and visualized with a fluorescence microscope (Life Technologies).

### Gelatin zymography

The culture medium from various pre-treated HTR8/SVneo cells was diluted with 4X sample buffer (8% SDS (w/v), 0.04% bromophenol blue (w/v), 0.25 M Tris), and incubated at 37 °C for 30 min. Equal amounts of protein were subjected to electrophoresis. Subsequently, renaturation buffer (2.5% Triton X-100 and 50 mM Tris–HCl (pH 7.5)) was used to wash the gels for 30 min at room temperature twice, followed by incubating the gels in a calcium assay buffer (50 mM Tris, 10 mM CaCl_2_, 1 mM ZnCl_2_, 1% Triton X-100, pH 7.5) at 37 °C overnight. Next, the gels were stained with Coomassie Brilliant Blue R250 at room temperature for 1 h and de-stained in 10% acetic acid (v/v). Finally, the gels were scanned by an image analyzer, the Quantity One System (Bio-Rad).

### Co-immunoprecipitation (Co-IP)

Co-IPs were performed according to a standard protocol using a Pierce co-IP Kit (ThermoFisher Scientific). Briefly, differently pre-treated cells were harvested in ice-cold IP Lysis/Wash Buffer, before centrifugation at 13 000 × *g* for 10 min to pellet the cell debris. Then, the IP was performed by the addition of antibodies to cell lysates: rabbit anti-AKT antibody (Cell Signaling Technology) to IP the SRC-3 protein, and rabbit anti-SRC-3 antibody (Cell Signaling Technology) to IP the AKT protein. All co-IP steps were performed at 4 °C unless otherwise indicated. Subsequently, protein A/G beads (Thermo Fisher Scientific) were added for an additional 2 h. The immunoprecipitated proteins were washed five times with IP Lysis/Wash Buffer. Finally, proteins were resolved by SDS/PAGE and immunoblotted with antibodies as indicated.

### Statistical analysis

All values are expressed as means ± standard deviation (SD). The data were analyzed using GraphPad Prism software (version 6.0, California, USA). Independent t-tests were used for intergroup comparisons of continuous variables. Statistical differences among multiple groups were evaluated by one-way analysis of variance (ANOVA), followed by the least significant difference multiple-comparisons test, as appropriate. P-values < 0.05 were considered to be statistically significant.

## Supplementary information

Supplementary info file

## Data Availability

Supporting data and essential materials for reproducibility of this study are available upon request made to the corresponding authors.
