# Supplementary material for: Hypoxia-induced Downregulation of SRC-3 Suppresses Trophoblastic Invasion and Migration Through Inhibition of the AKT/mTOR Pathway: Implications for the Pathogenesis of Preeclampsia
Source: Sci Rep. 2019 Jul 17;9:10349. doi: 10.1038/s41598-019-46699-3 (PMC6637123; doi:10.1038/s41598-019-46699-3)
Supplement: Supplementary file 1 — Supplementary info file [file 41598_2019_46699_MOESM1_ESM.pdf]

Hypoxia-induced Downregulation of SRC-3 Suppresses Trophoblastic Invasion and Migration Through Inhibition of the AKT/mTOR Pathway: Implications for the Pathogenesis of Preeclampsia

Chengjin He[1,2,3#], Nan Shan[1,2,3#], Ping Xu[1,2,3], Huisheng Ge[1,2,3], Yu Yuan[1,2,3], Yangming Liu[1,2,3], Pu Zhang[4], Li Wen[1,2,3], Fumei Zhang[1,2,3], Liling Xiong[1,2,3], Chuan Peng[3], Hongbo Qi[1,2,3*], Chao Tong[1,2,3*], Philip N. Baker[2,5,6]

[1] Department of Obstetrics, The First Affiliated Hospital of Chongqing Medical University, Chongqing 400016, China;

[2] International Collaborative Joint Laboratory of Reproduction and Development, Ministry of Education of China, Chongqing Medical University, Chongqing 400016, China;

[3] State Key Laboratory of Maternal and Fetal Medicine of Chongqing Municipality, The First Affiliated Hospital of Chongqing Medical University, Chongqing 400016, China;

[4] College of Pharmacy, Chongqing Medical University, Chongqing 400016, China

[5] Liggins Institute, University of Auckland, Auckland 1142, New Zealand;

[6] College of Medicine, Biological Sciences and Psychology, University of Leicester, Leicester LE1 7RH, UK.

# These authors contributed equally to this work.

*Correspondence to Prof. Chao Tong and Prof. Hongbo Qi.

Address: Department of Obstetrics, The First Affiliated Hospital of Chongqing Medical University, 1 Youyi Road, Yuzhong District, Chongqing 400016, China

Tel: +86 23 89011101 (H.Q); +86 23 89011865 (C.T).

Fax: +86 23 89011102 (H.Q); +86 23 89011791(C.T);

Email: chaotongcqmu@163.com (C.T); qihongbo728@163.com (H.Q)

Supplementary Figures

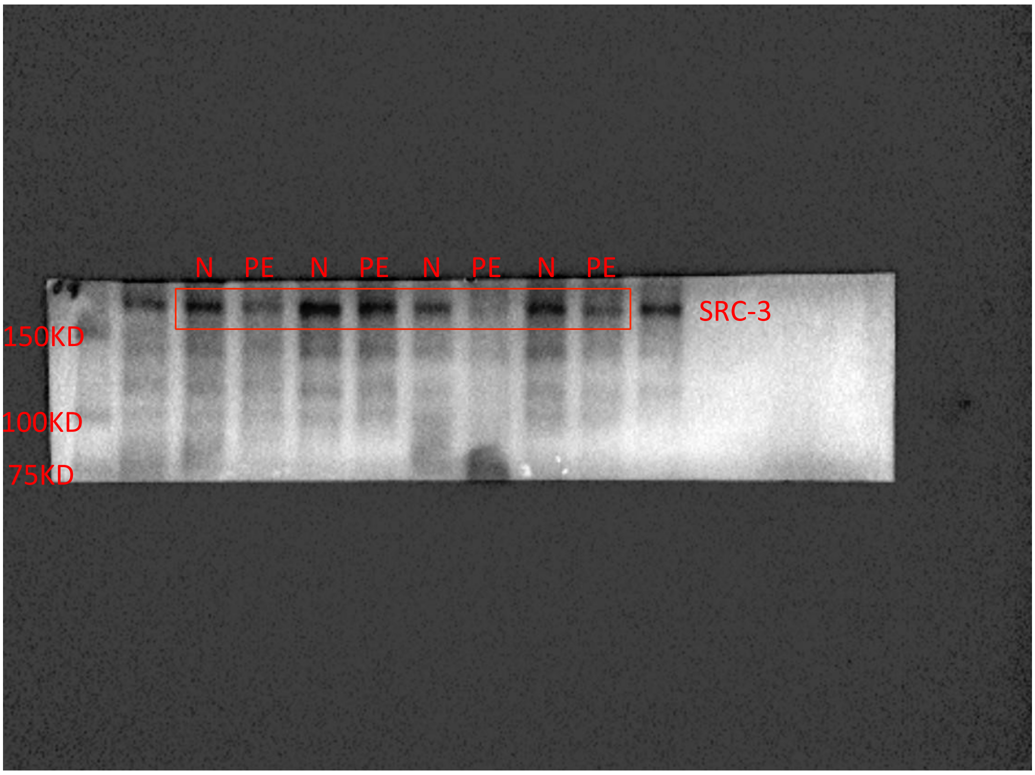

Fig. S1. Full-length blots of SRC-3 in Fig.1B.

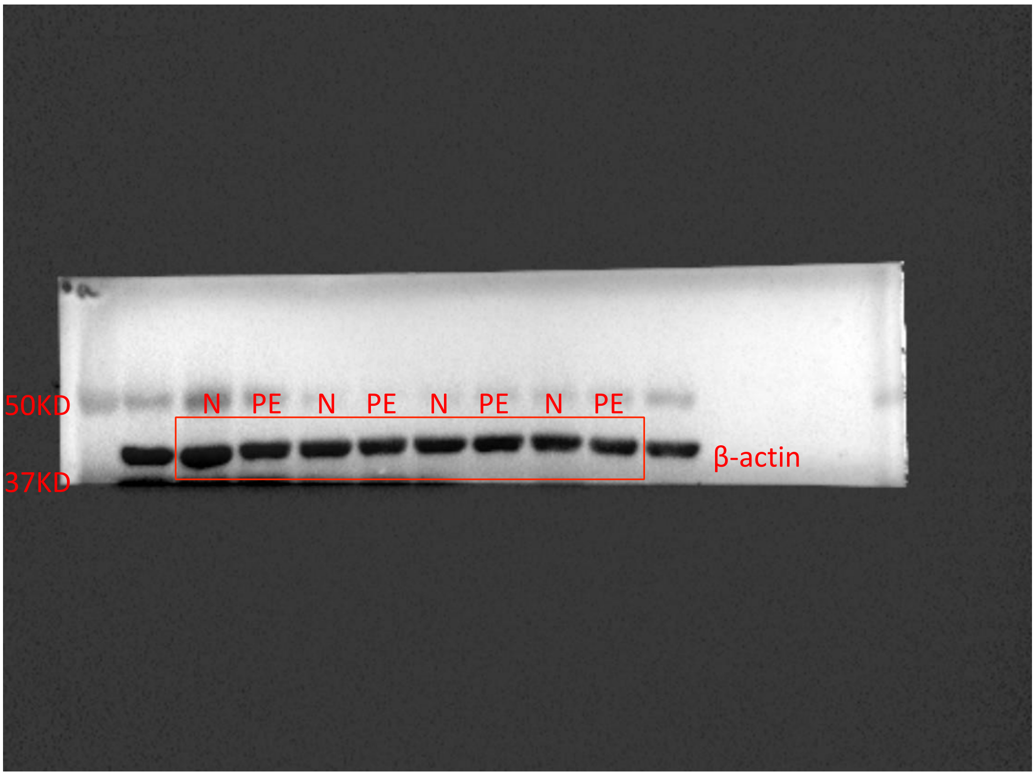

Fig. S2. Full-length blots of β-actin in Fig.1B.

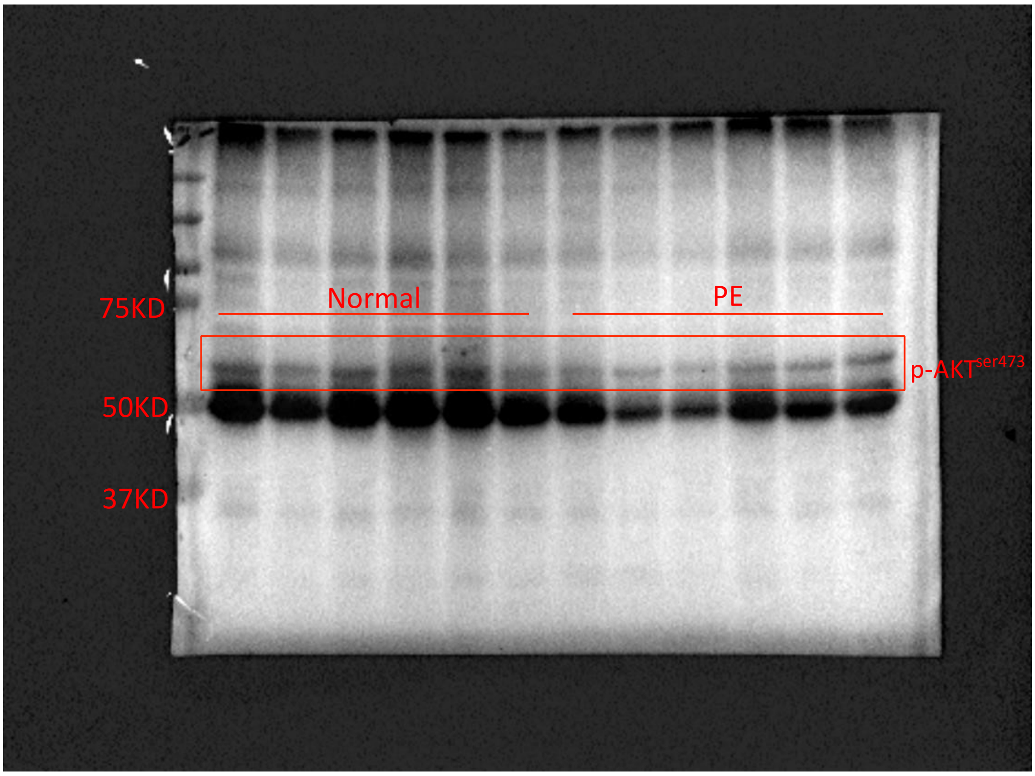

Fig. S3. Full-length blots of p-AKT^{ser473}in Fig.1C.

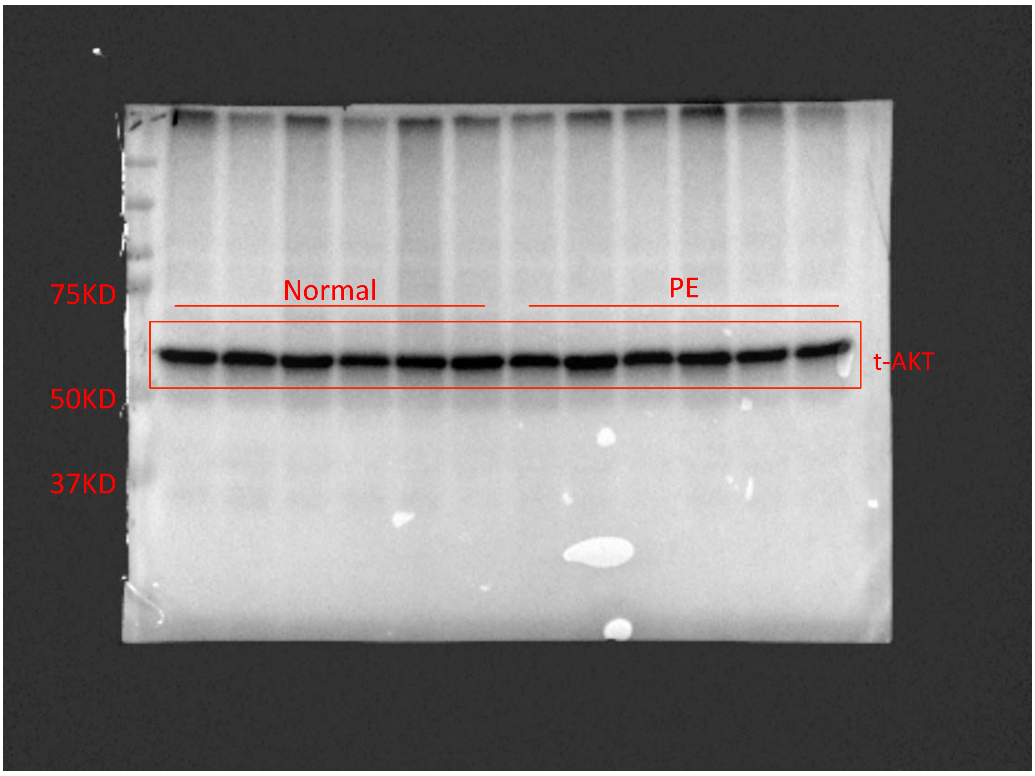

Fig. S4. Full-length blots of t-AKT in Fig.1C.

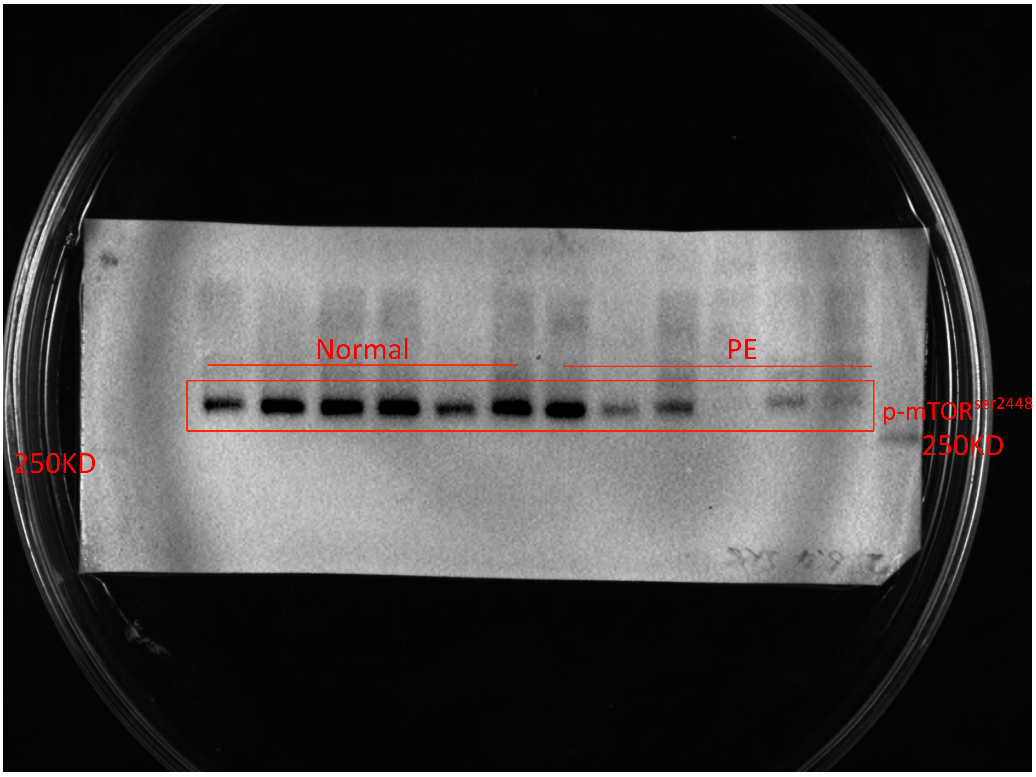

Fig. S5. Full-length blots of p-mTOR^{ser2448}in Fig.1C.

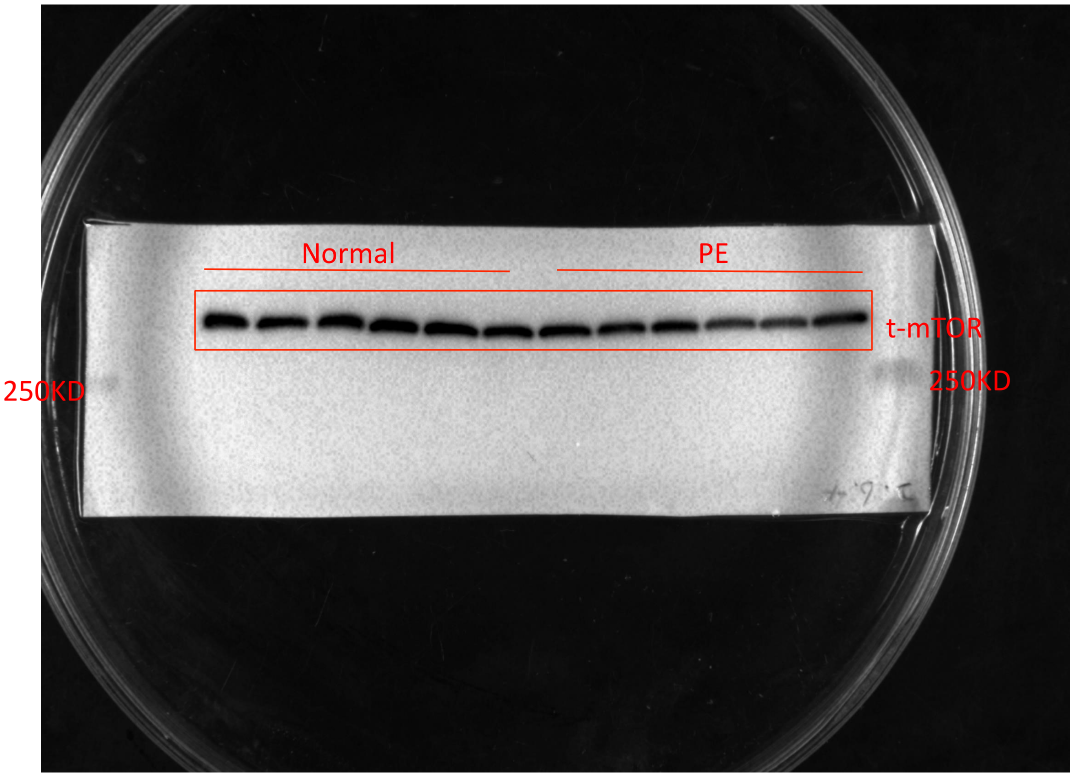

Fig. S6. Full-length blots of t-mTOR in Fig.1C.

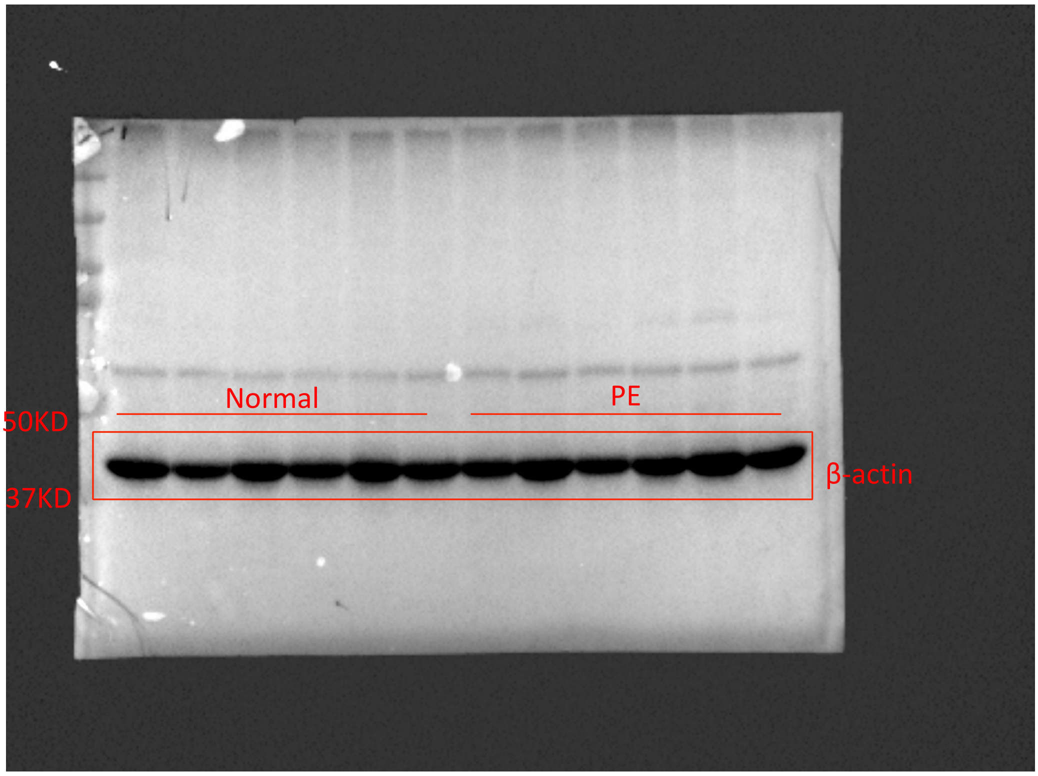

Fig. S7. Full-length blots of β-actin in Fig.1C.

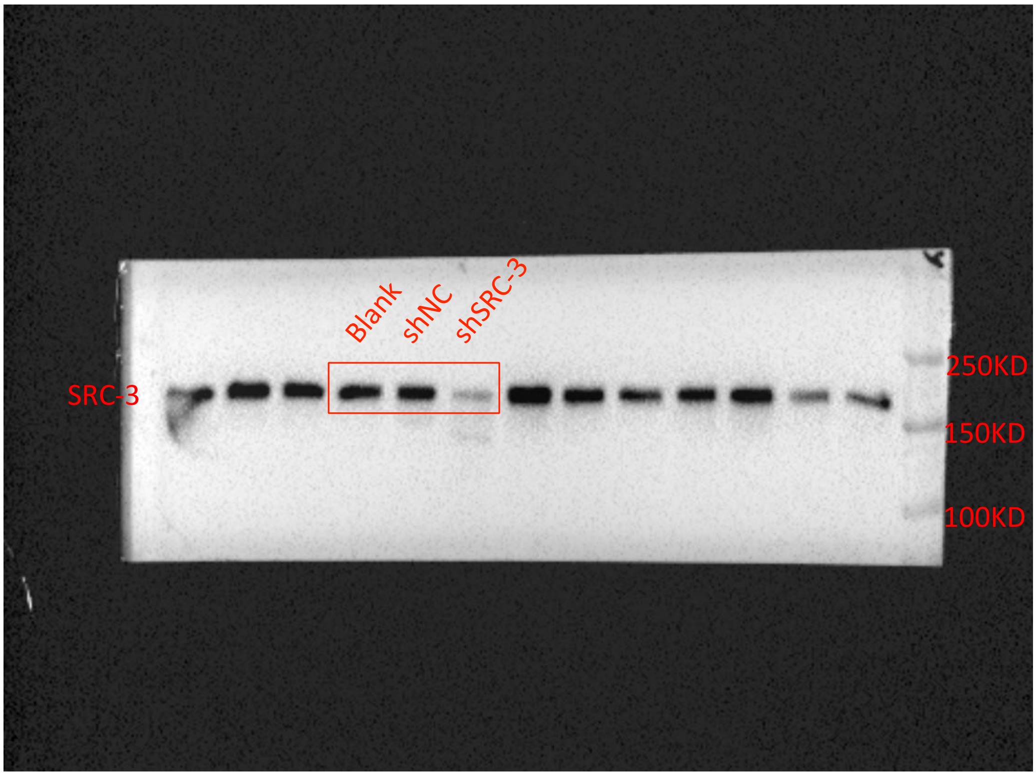

Fig. S8. Full-length blots of SRC-3 in Fig.2B.

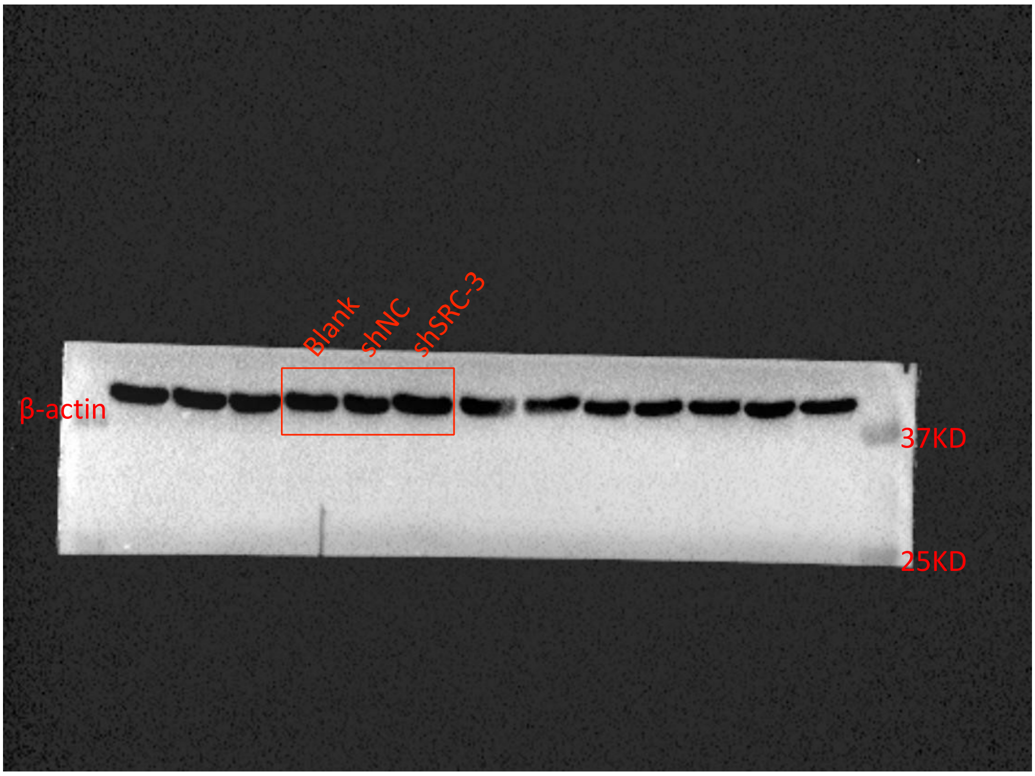

Fig. S9. Full-length blots of β-actin in Fig.2B.

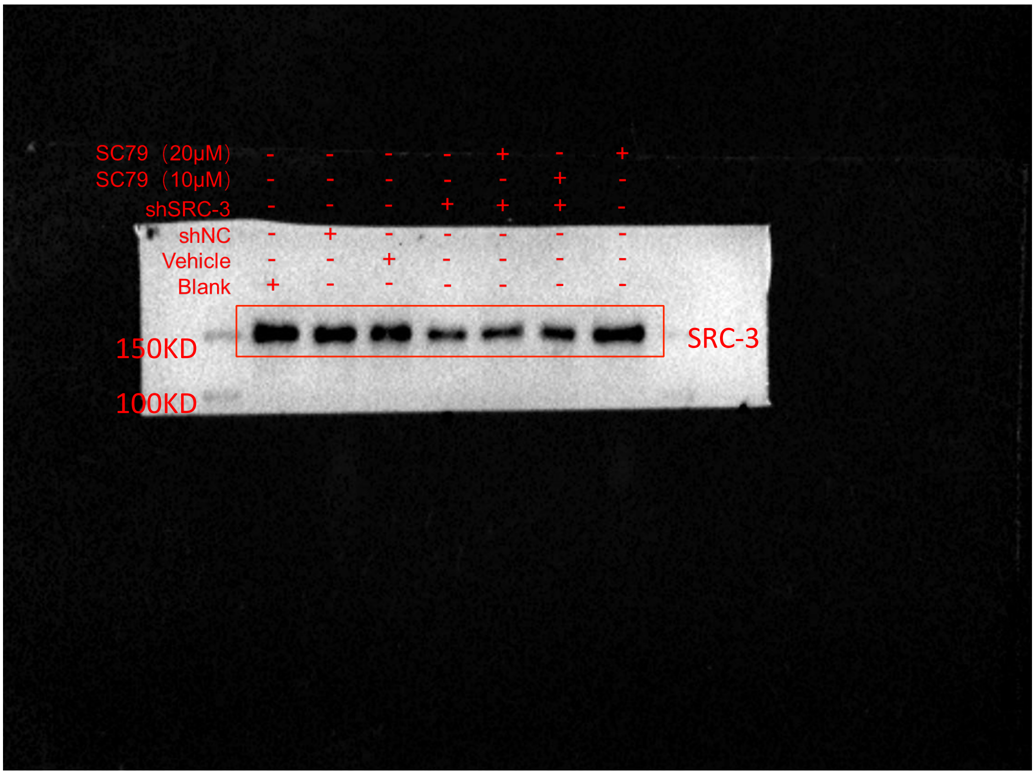

Fig. S10. Full-length blots of SRC-3 in Fig.3C.

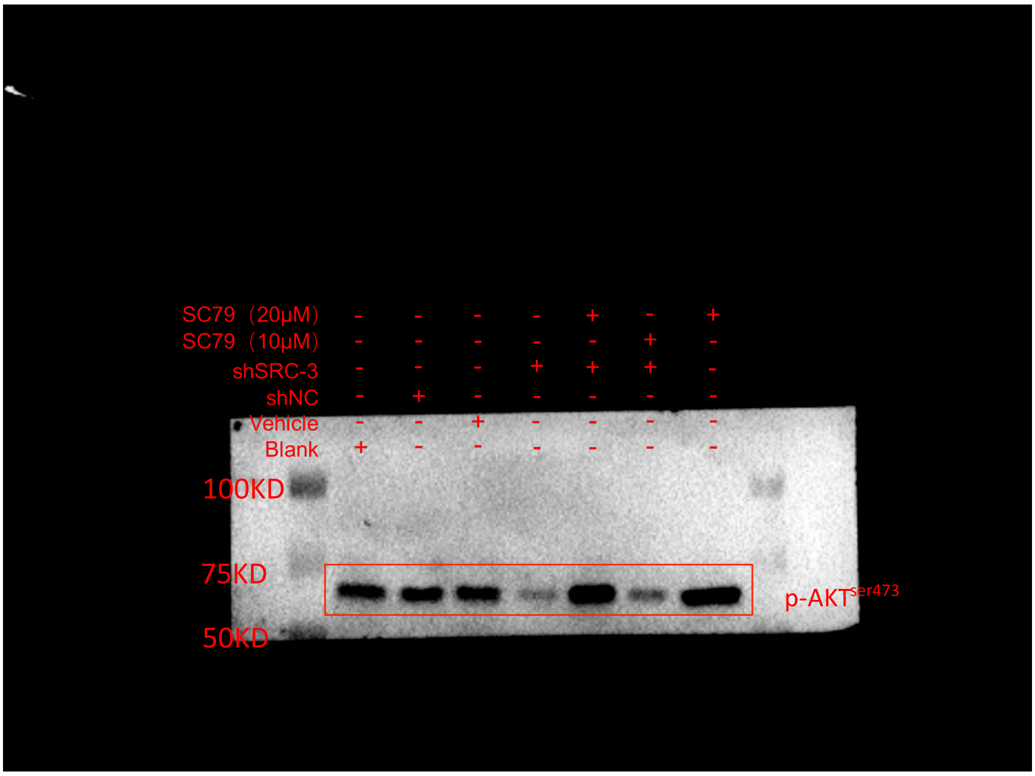

Fig. S11. Full-length blots of p-AKT^{ser473} in Fig.3C.

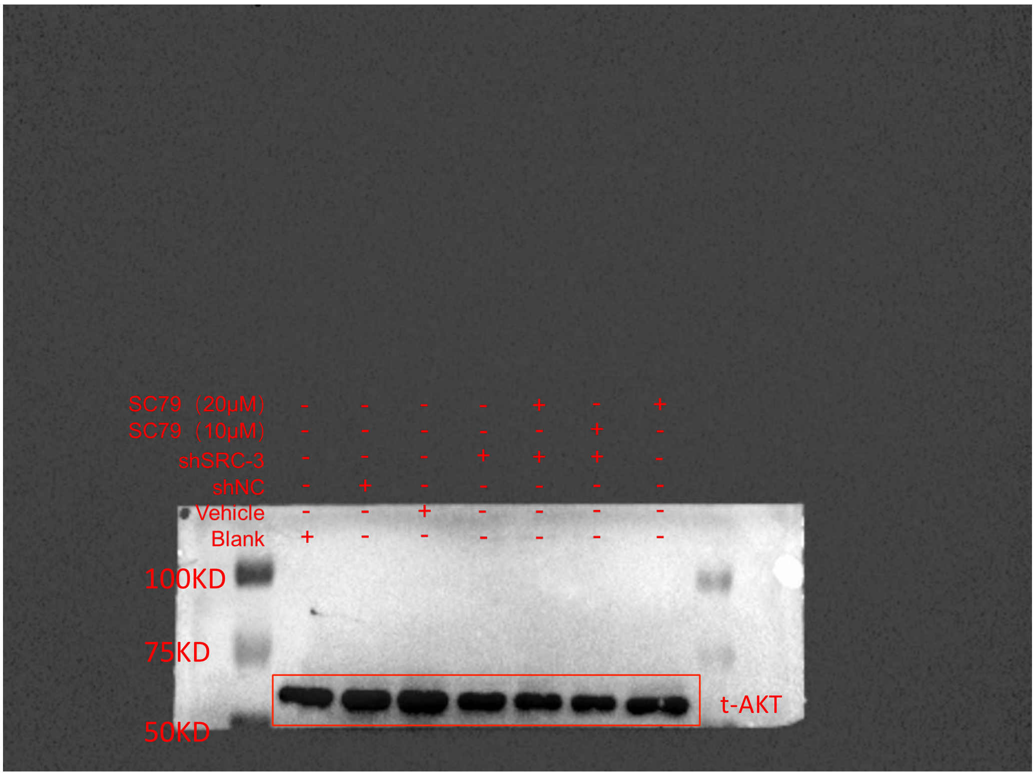

Fig. S12. Full-length blots of t-AKT in Fig.3C.

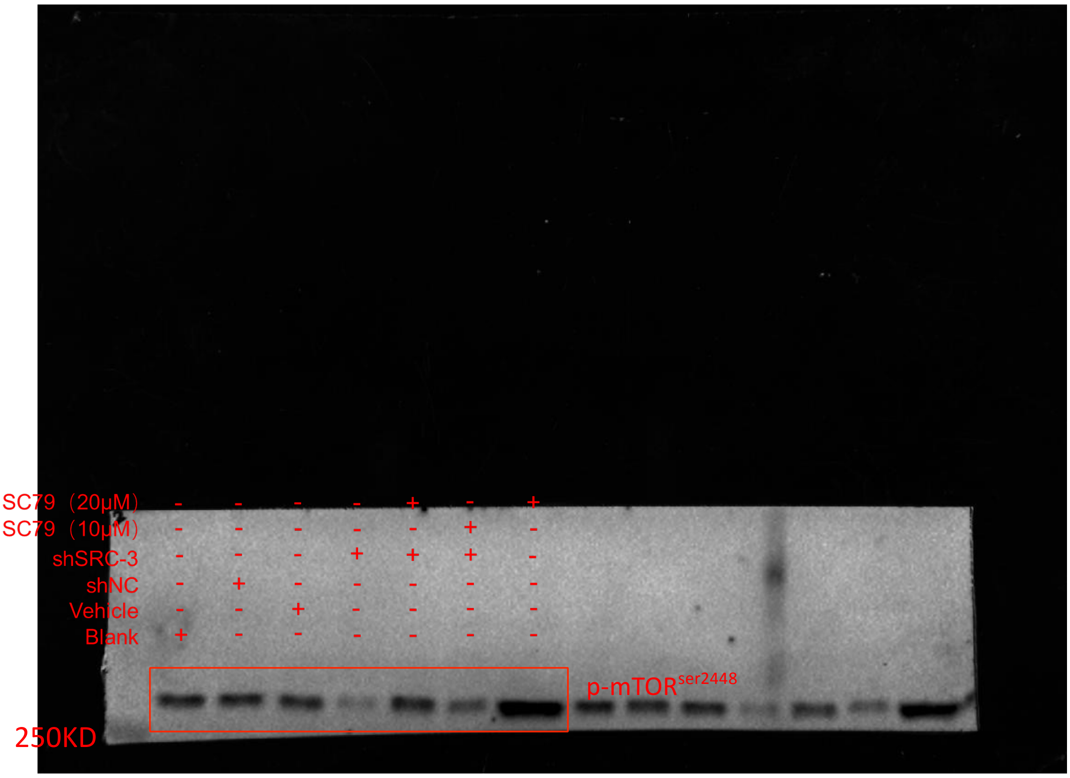

Fig. S13. Full-length blots of p-mTOR^{ser2448} in Fig.3C.

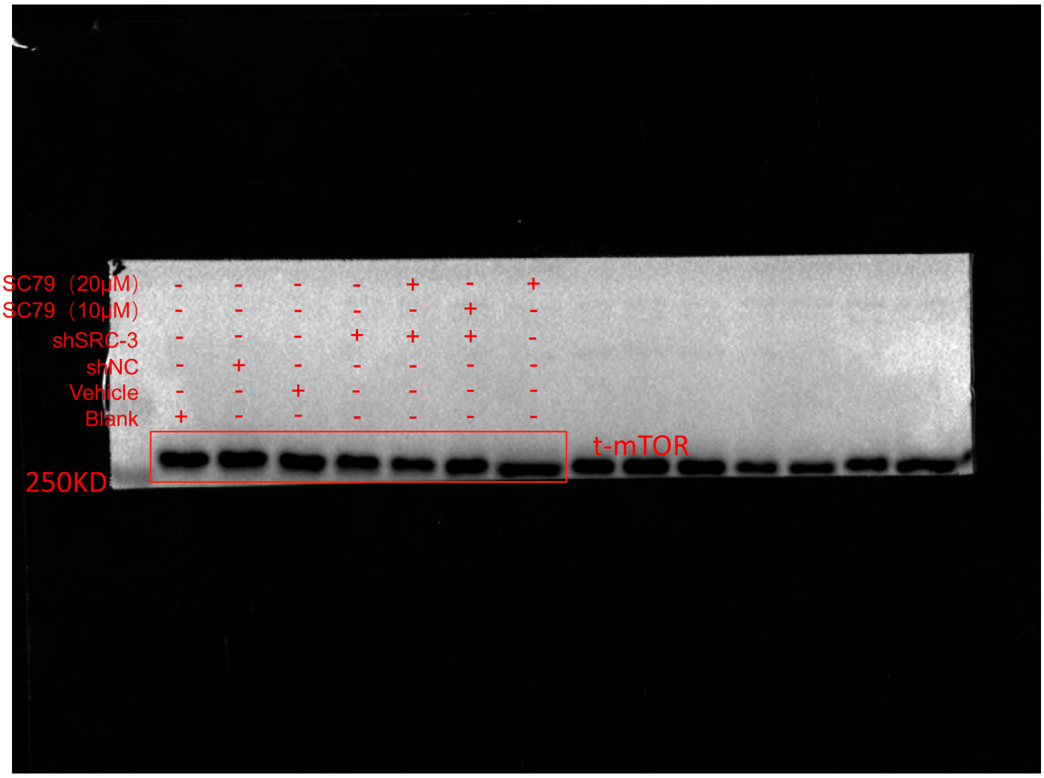

Fig. S14. Full-length blots of t-mTOR in Fig.3C.

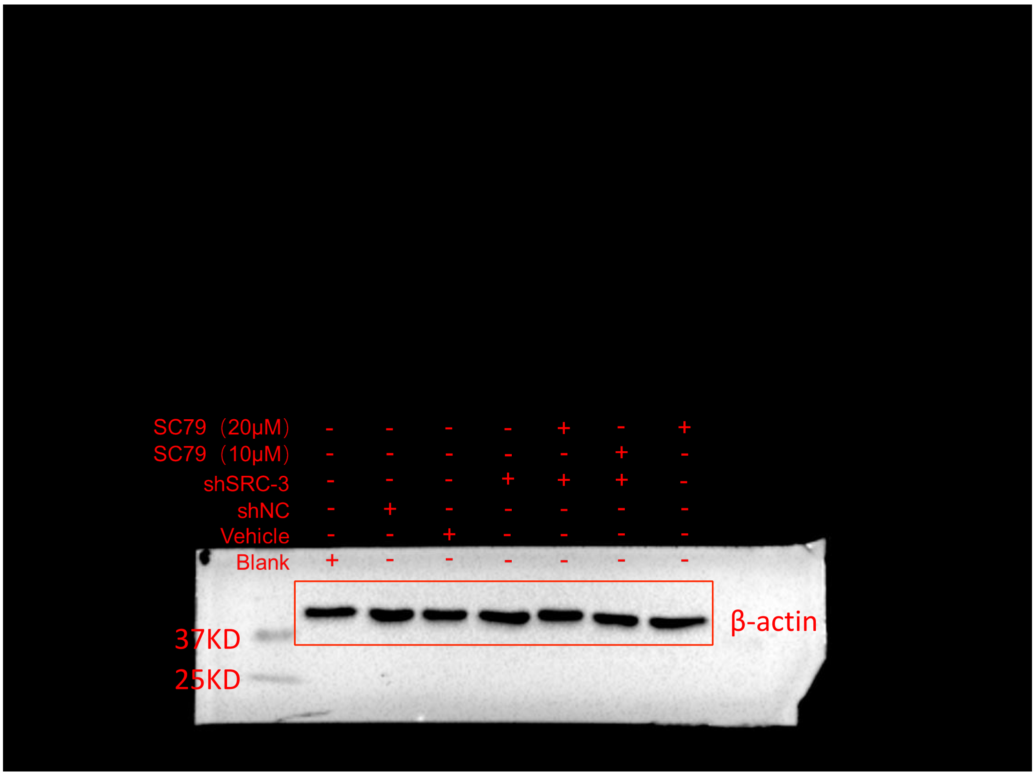

Fig. S15. Full-length blots of β-actin in Fig.3C.

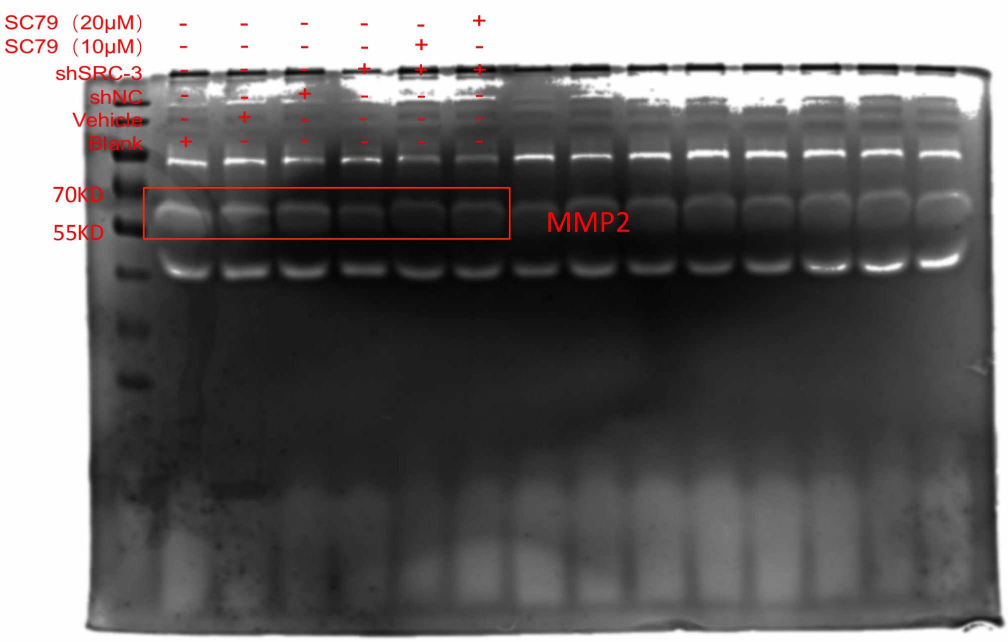

Fig. S16. Full-length gels of MMP2 in Fig.3D.

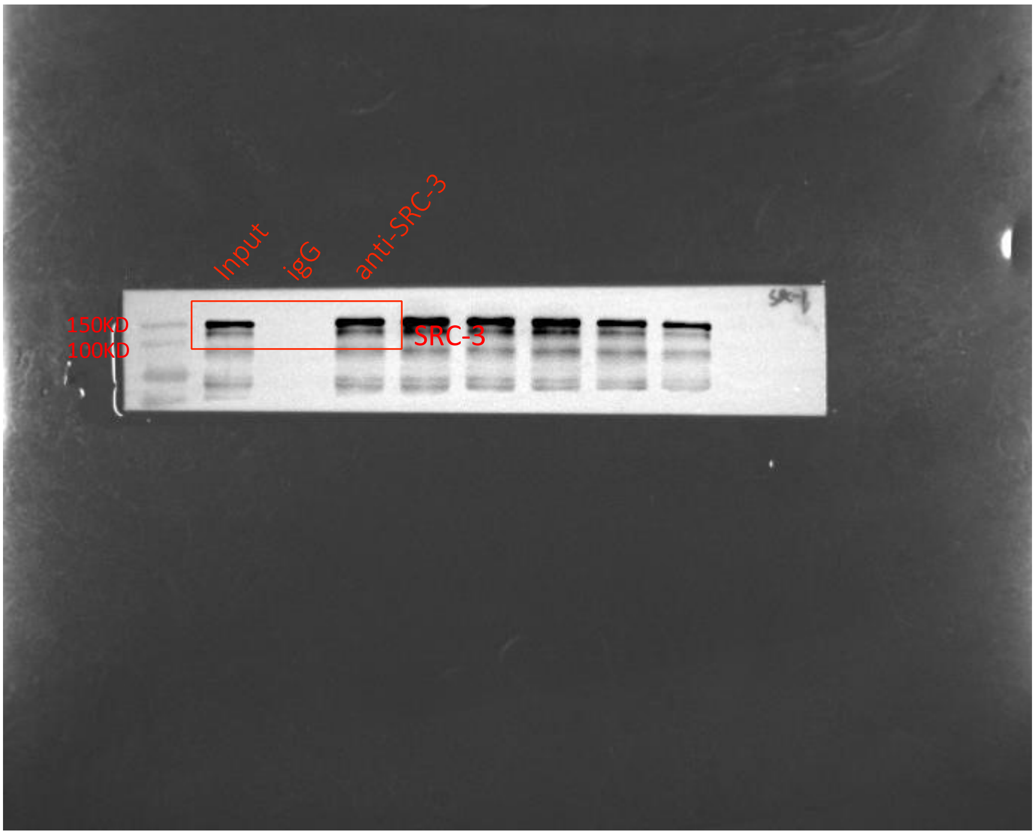

Fig. S17. Full-length blots of SRC-3 (upper panel) in Fig.4B.

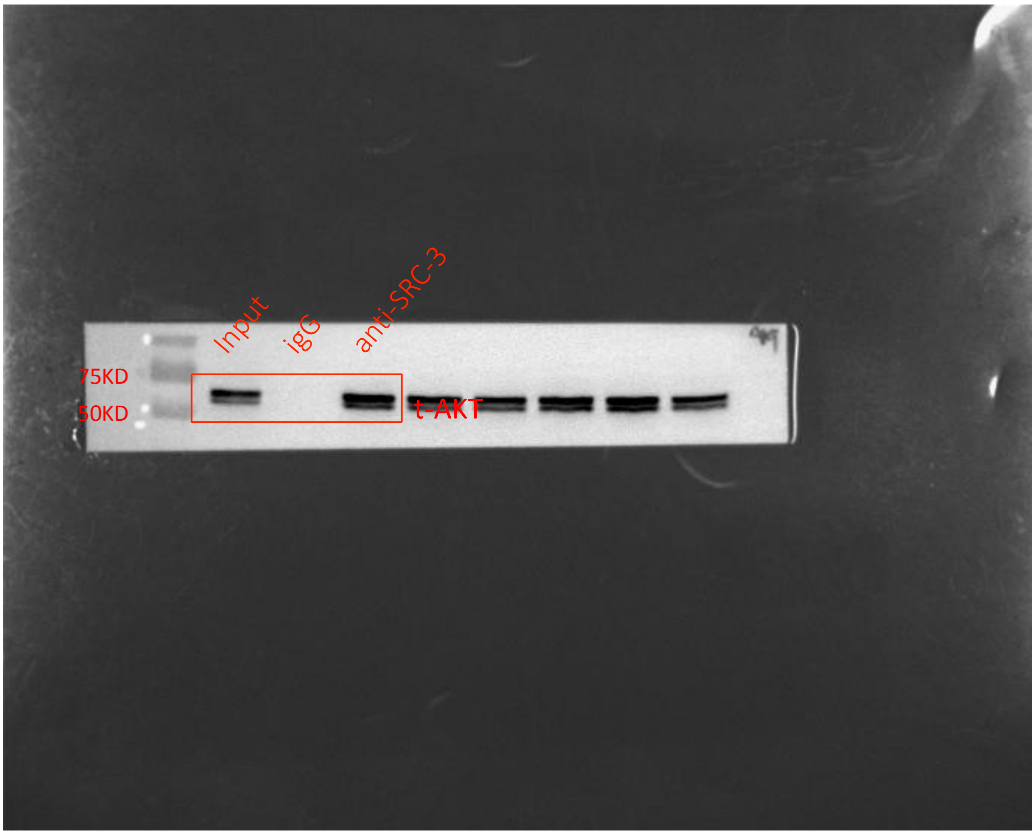

Fig. S18. Full-length blots of AKT (upper panel) in Fig.4B.

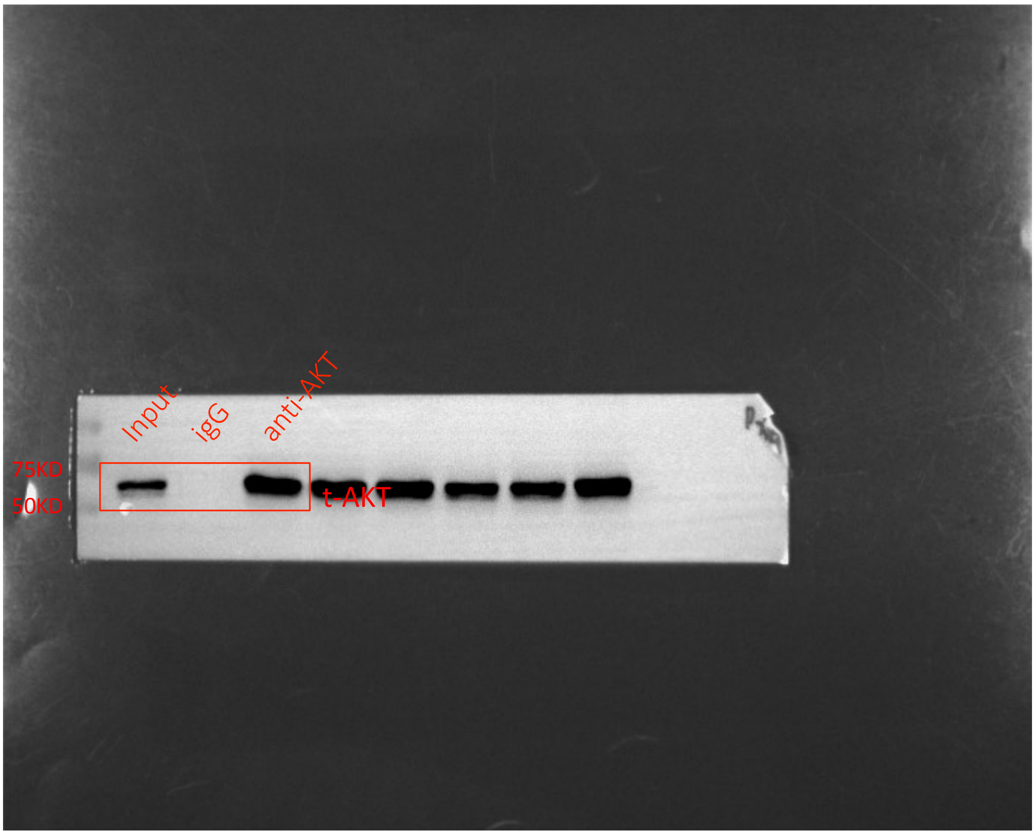

Fig. S19. Full-length blots of AKT (lower panel) in Fig.4B.

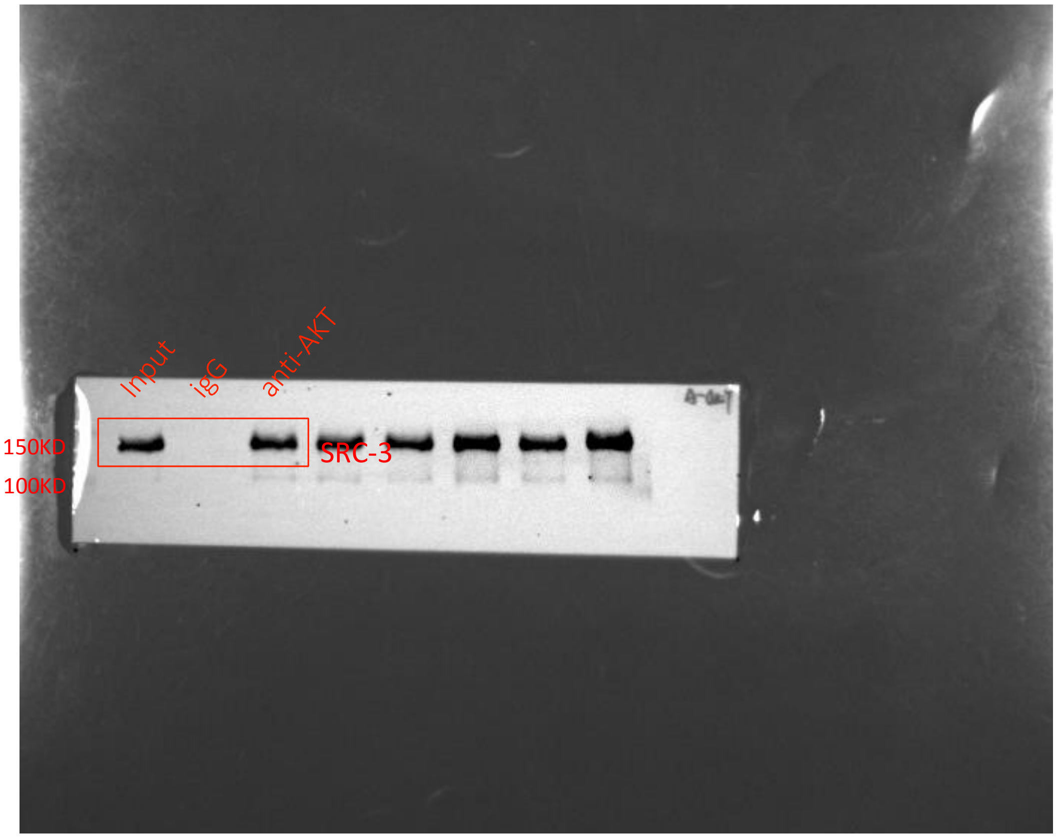

Fig. S20. Full-length blots of SRC-3 (lower panel) in Fig.4B.

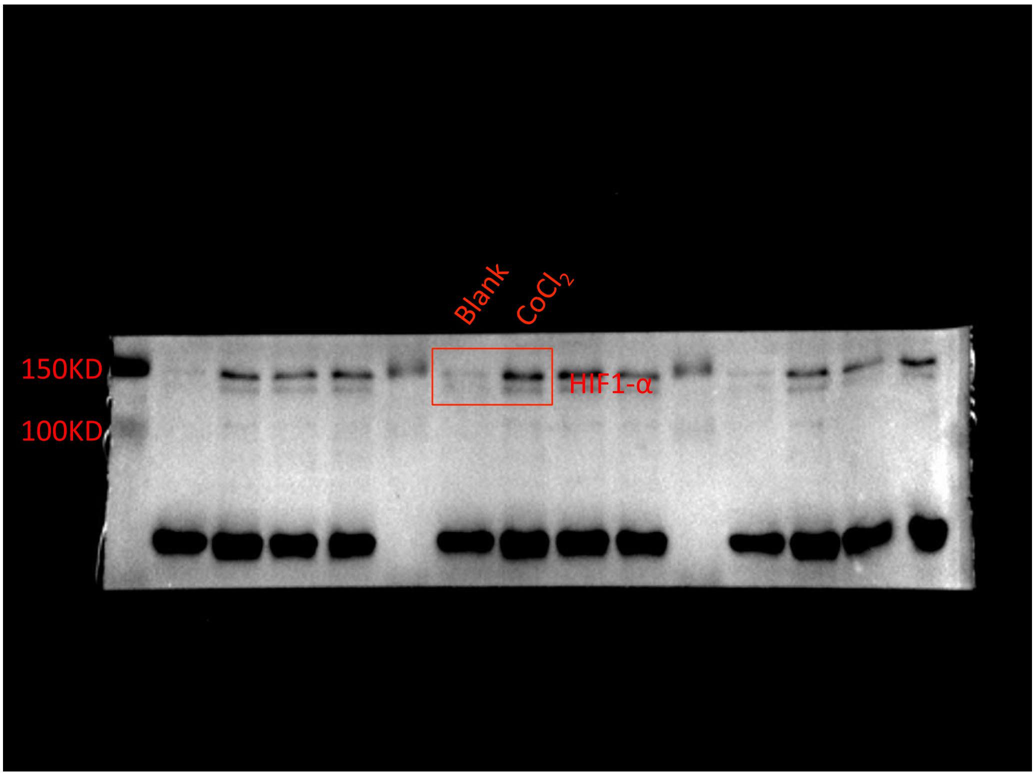

Fig. S21. Full-length blots of HIF1-α in Fig.5A.

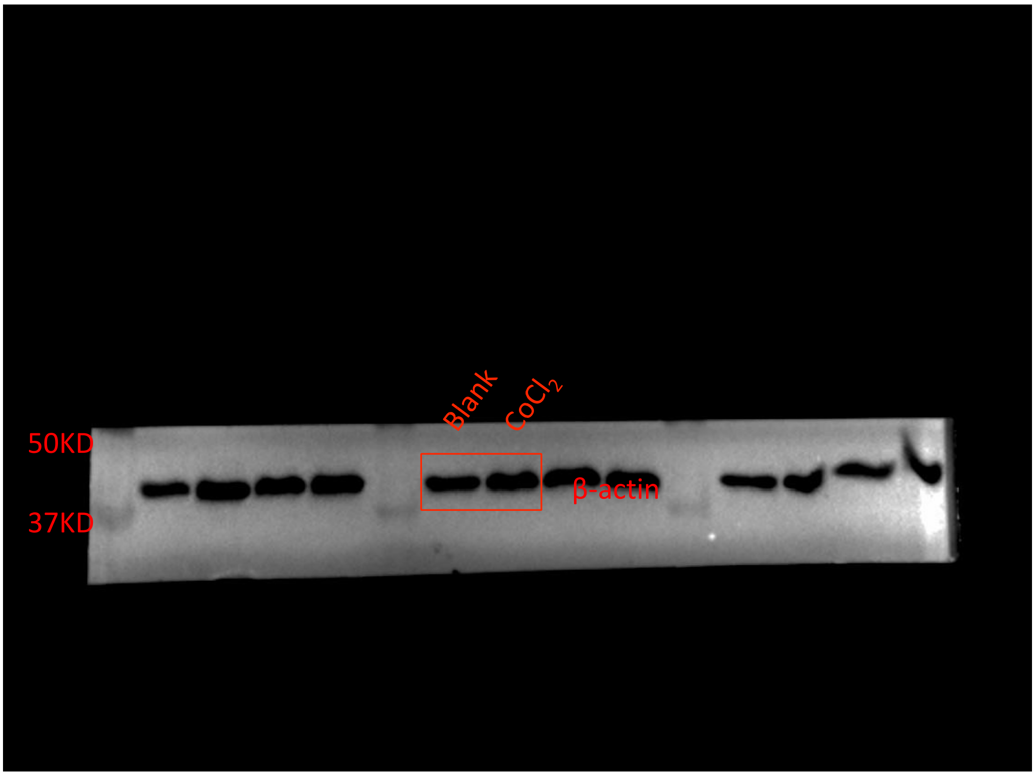

Fig. S22. Full-length blots of β-actin in Fig.5A.

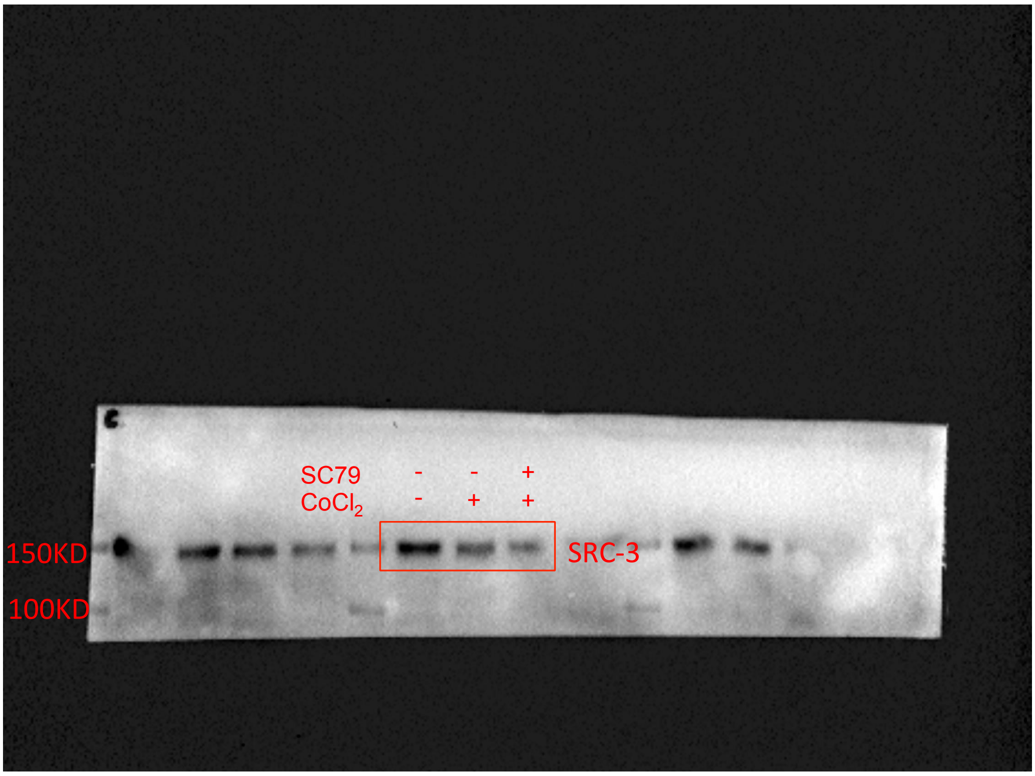

Fig. S23. Full-length blots of SRC-3 in Fig.5C.

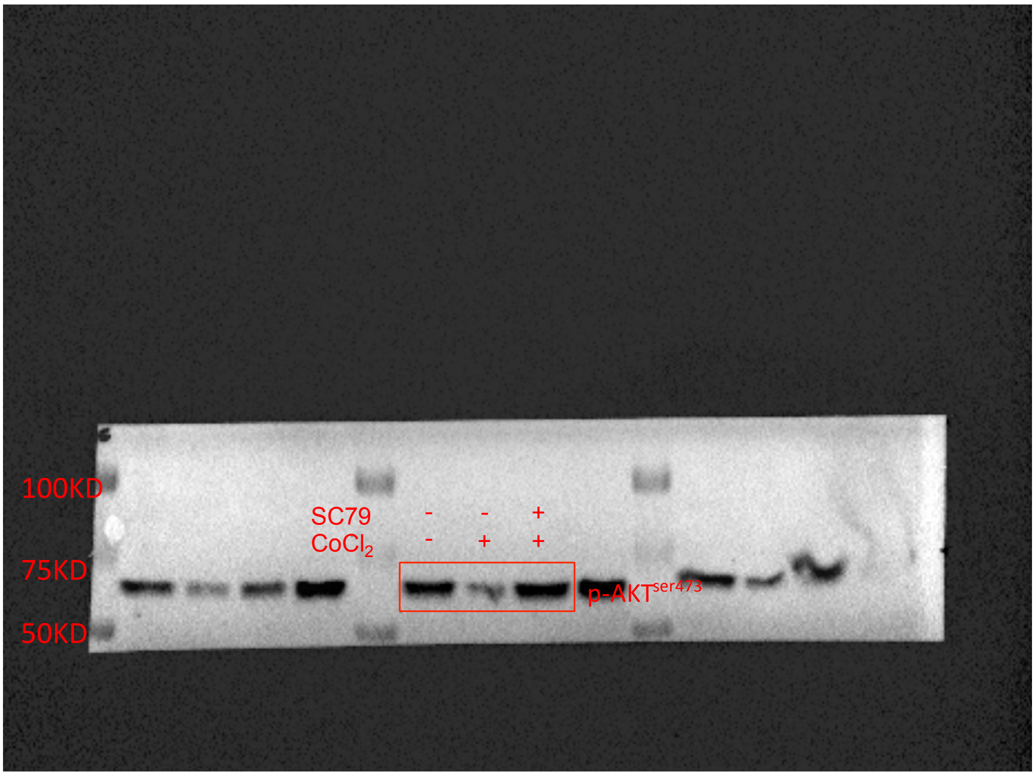

Fig. S24. Full-length blots of p-AKT^{ser473} in Fig.5C.

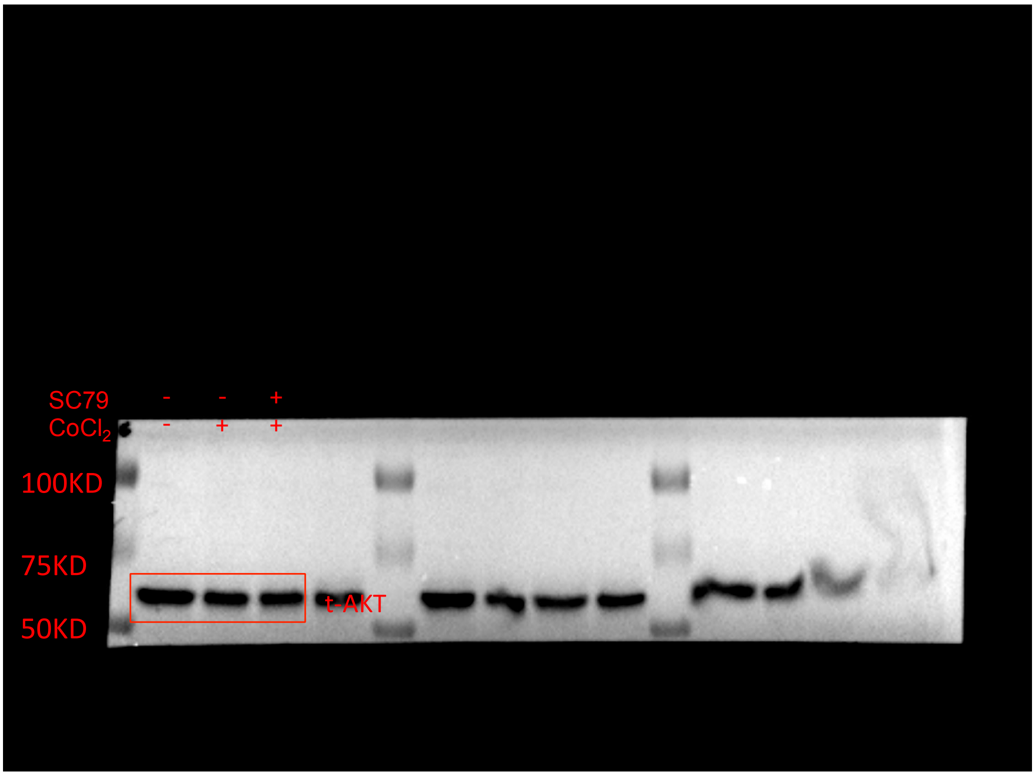

Fig. S25. Full-length blots of t-AKT in Fig.5C.

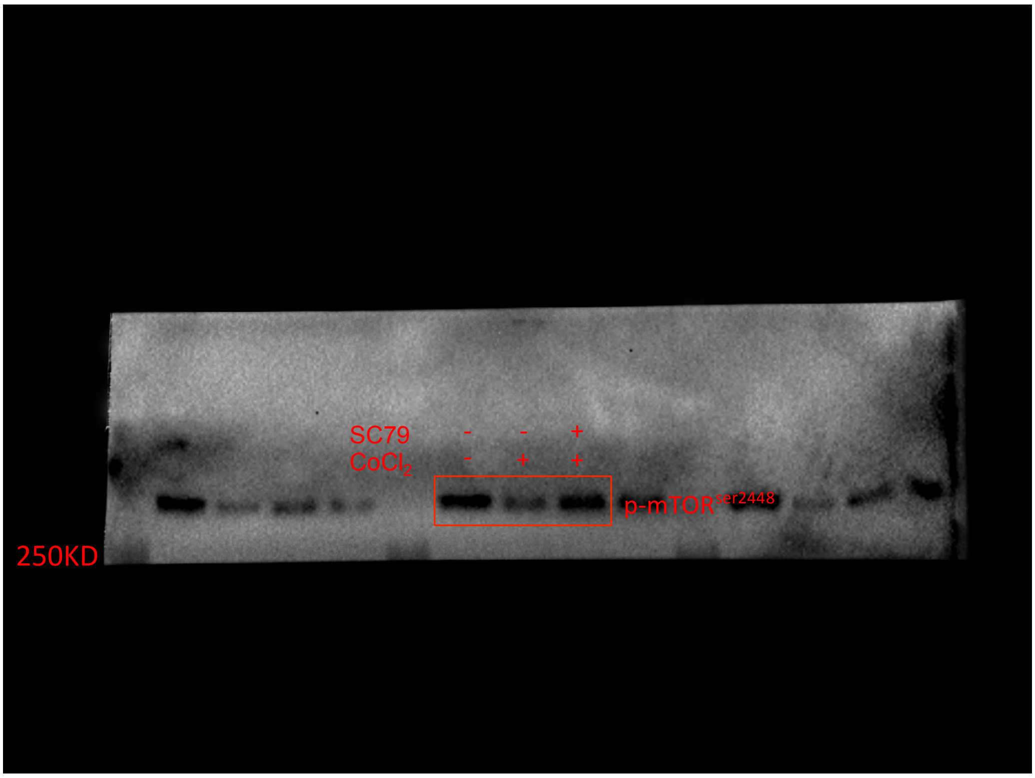

Fig. S26. Full-length blots of p-mTOR^{ser2448} in Fig.5C.

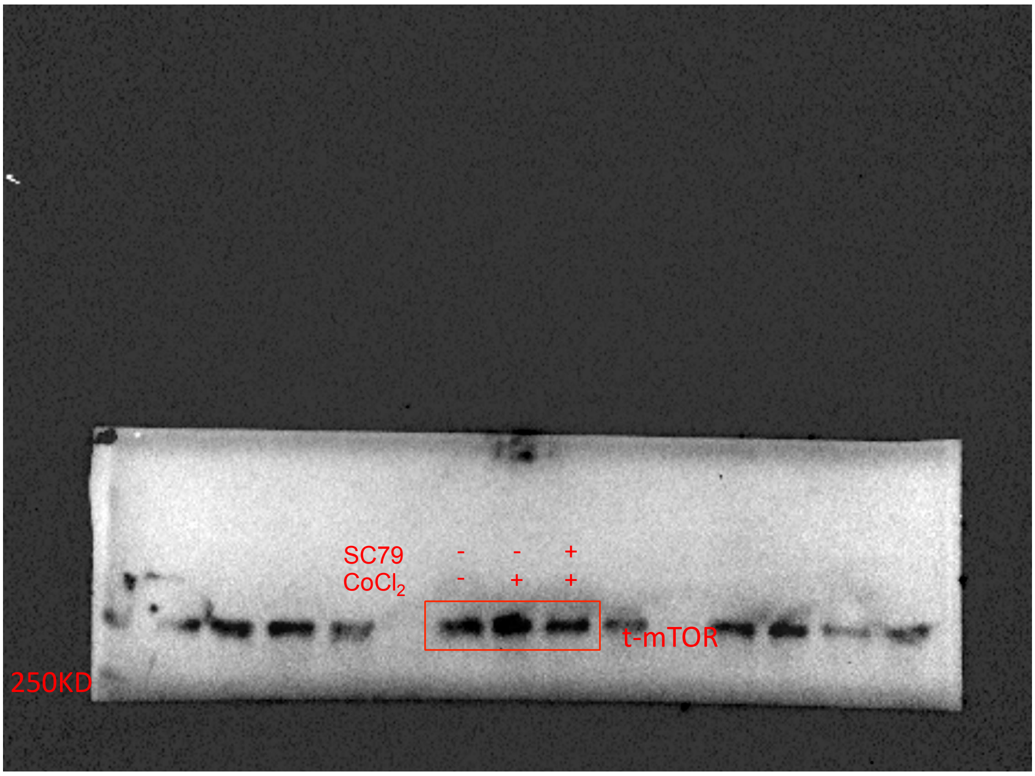

Fig. S27. Full-length blots of t-mTOR in Fig.5C.

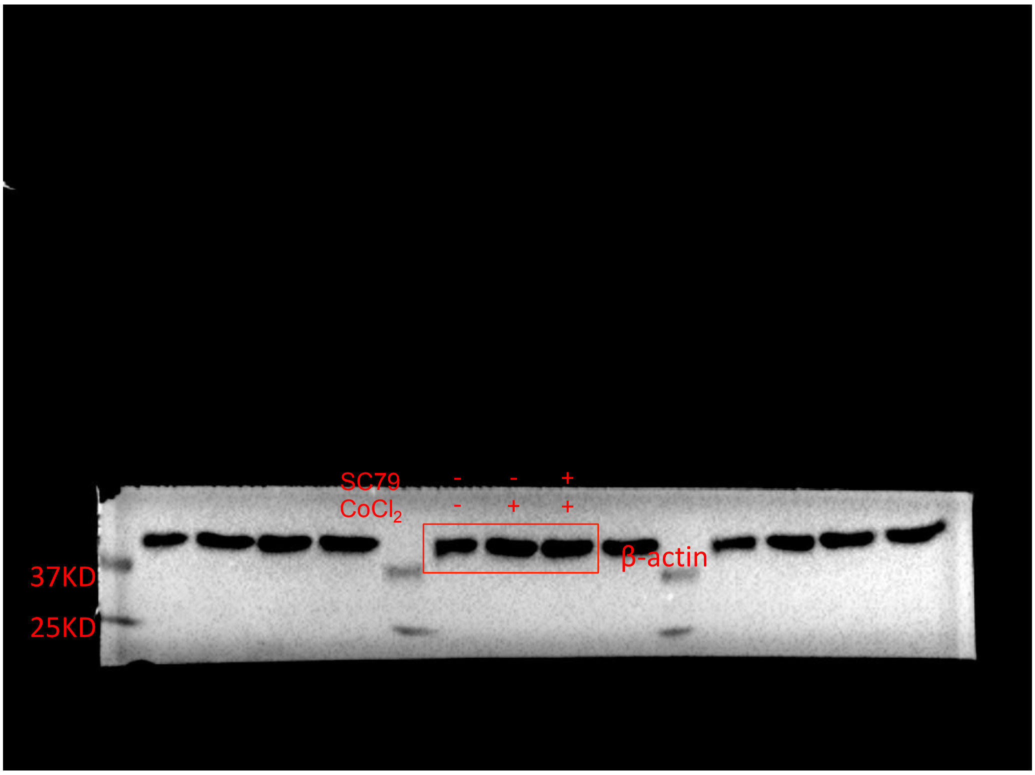

Fig. S28. Full-length blots of β-actin in Fig.5C.

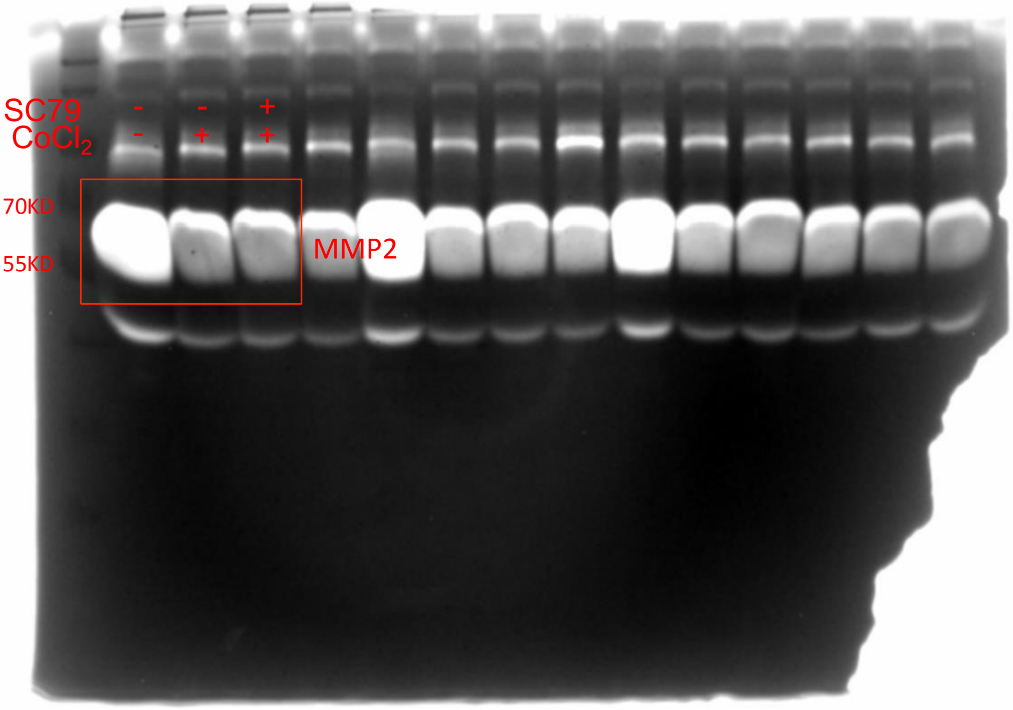

Fig. S29. Full-length gels of MMP2 in Fig.5F.
